# DOGMA: *de novo* assembly of densely labelled optical DNA maps using a matrix profile approach

**DOI:** 10.1371/journal.pone.0335633

**Published:** 2025-12-01

**Authors:** Albertas Dvirnas, Luis Mario Leal-Garza, Zahra Abbaspour, Erik Fröbrant, Karolin Frykholm, Marie Wrande, Linus Sandegren, Fredrik Westerlund, Tobias Ambjörnsson

**Affiliations:** 1 Centre for Environmental and Climate Science, Lund University, Lund, Sweden; 2 Department of Life Sciences, Chalmers University of Technology, Gothenburg, Sweden; 3 Department of Astronomy and Theoretical Physics, Lund University, Lund, Sweden; 4 Centre for Antibiotic Resistance Research in Gothenburg (CARe), Gothenburg, Sweden; 5 Department of Medical Biochemistry and Microbiology, Uppsala University, Uppsala, Sweden; Fisheries and Oceans Canada, CANADA

## Abstract

In optical genome mapping (OGM), large numbers of individual DNA maps—sequence-specific data series along single DNA molecules—are produced. Such individual maps have to be stitched together in a process called *de novo* OGM assembly in order to create consensus OGM maps for corresponding regions along the chromosomes. While there are several types of experimental OGM assays, not all of them have *de novo* OGM assembly tools available. In particular, in densely-labelled OGM there are no such tools. Here, we present and evaluate DOGMA, a *de novo* OGM assembly algorithm for densely labelled OGM data which uses matrix profiles. Matrix profile has transformed how data mining problems are approached in time series analysis. Yet, this algorithm has not been widely explored outside of the time series community— we here use it for OGM *de novo* assembly for the first time. Further novelties in our algorithm are the introduction of two scores for each individual alignment, use of p-values, a visual representation as barcode islands and the introduction of a method for generating consensus barcodes using amplitude adjustment. Utilizing p-values helps mitigate the risk of errors in the assemblies as caused by false positives. We demonstrate our algorithm by applying it for *de novo* OGM assembly of synthetic datasets and of an experimental dataset from an *Escherichia coli* genome. We validate the assemblies using corresponding reference genomes and investigate the strengths and limitations of the algorithm. *De novo* OGM assembly of dense optical DNA maps shows promise as a complement or an alternative to current OGM techniques for other types of genome mapping assays. The code is available at: https://github.com/dnadevcode/dogma.

## Introduction

Optical DNA mapping (ODM) is an umbrella term for methods that visualize sequence information on single DNA molecules on the 10-1000 kilobase pairs (kb) length scale [1]. ODM involves labelling, stretching, and imaging of intensity profiles along individual DNA molecules using a fluorescence microscope. Optical DNA mapping is a promising technique for addressing challenges within this size range because it can potentially bridge the gap between traditional methods such as Next Generation Sequencing (NGS) and Fluorescence in situ Hybridization (FISH).

Optical Genome Mapping (OGM), a genome-wide application of optical DNA mapping, has been used for detecting long-range structural variations, aiding DNA sequence assembly of complex genomes [2–4] and, in combination with other labeling schemes, mapping epigenetic marks [5,6] and DNA damage across genomes [7]. Consequently, OGM holds potential as a future complement or even replacement for traditional techniques in these specific applications.

While OGM hold promise for bridging the size gap inherent in other DNA analysis technologies, a current limitation is that during preprocessing DNA molecules get fragmented when extracted from cells due to shearing stress exerted by mechanical processes such as pipetting, vortexing, and shaking. In effect, the intensity profiles from single (fragmented) DNA molecules used in ODM may not cover the complete genome. One important theoretical challenge in the field is therefore to assemble intensity profiles for DNA fragments into a complete optical genomic profile—*de novo* OGM assembly (not to be confused with *de novo* assembly of DNA sequences in NGS). This challenge is addressed in this study.

To create the optical DNA maps, DNA needs to be labelled in a sequence-specific fashion. Depending on the labelling method OGM can be divided into two main categories, sparsely labelled OGM and densely labelled OGM [8]. In sparsely labelled OGM, the DNA backbone is detected by the use of a DNA binding fluorescent dye, and sequence specific motifs are labelled with a different color by the use of an enzyme. When the labelling is too dense to consider individual labels, one needs to resort to methods for the densely labelled OGM data series (herein, called barcodes). OGM based on sparse labelling has been commercialized by Bionano Genomics and is already extensively used in, for example, cancer diagnostics. In contrast, dense labelling schemes have been much less explored, but have certain traits that make them interesting as a complement or substitute to sparse labelling. Important advantages include a simpler sample preparation protocol without enzymatic reactions and that a single emission color is enough to produce the barcode. The latter means that dense labelling schemes hold promise for OGM protocols in a cheap, single color instrument, but also that it can be combined with sparse labelling to increase the information density along each DNA molecule.

During the last decade, we have developed an OGM protocol for dense labelling that is based on competitive binding of the fluorescent molecule YOYO-1 and the AT-specific antibiotic netropsin to DNA. As netropsin binds preferentially to AT-rich DNA, it hinders YOYO-1 from binding those regions, which leads to DNA molecules with an emission intensity variation along the backbone that reflects the underlying local GC content. This protocol has been applied to various studies, including cultivation-free typing of bacteria [9,10], rapid tracing of resistance plasmids [11] as well as enzyme-free mapping of the human genome [7] and detecting structural variations [12].

Here, we tackle the problem of *de novo* OGM assembly of densely labelled barcodes. There are no current dedicated tools to perform and visualize a *de novo* assembly of barcodes (in contrast, OGM assembly for sparsely labelled OGM has been extensively studied before [1,13–15]). One previous algorithm, which uses continuous information for sparsely labelled OGM data, could potentially be used for the type of data considered herein [14]. However, that algorithm is only scalable with a fast pre-alignment step [16].

Our OGM assembly method is made computationally efficient by making use of Matrix profile (MP) algorithms. MP has advanced the approach for solving various data mining problems in time series analysis since its inception [17,18]. It provides algorithms to efficiently compare sub-timeseries between datasets. MP is computationally more efficient than similar algorithms used previously in the OGM field [19]. We have previously developed a method to estimate the significance of MP scores for dense OGM [12].

In this paper, we, for the first time, present a pipeline for *de novo* OGM assembly of densely labelled OGM barcodes that we refer to as Dense Optical Genome Mapping Assembly (*DOGMA*). We use inspiration from the sparse labelling algorithms [13,20], which we extend to a dense labelling setting. In NGS, there are two main approaches to perform sequence assemblies, the overlap-layout-consensus (OLC) [21] or De-Brujn graph (DBG) approach [22], and our OGM assembly method is a version of the OLC approach. We use MP for fast computation of overlap scores between barcodes. We estimate the overlap significance using a previously developed null model with pre-generated model constants. Furthermore, as an output our algorithm provides visually informative contiguous regions (so called barcode islands), which are easily interpretable. We also provide a method for calculating consensus barcodes from the barcode islands by introduction of a method for amplitude adjustment. We test our algorithm both on synthetic data with varying levels of noise, and on real ODM data from a bacterial genome. The strength of our method is that we are able to create large barcode islands, that the method is robust and that we have good control of the false positives.

## Materials and methods

### Overview of the *de novo* OGM assembly

Here, we introduce DOGMA, a method for *de novo* OGM assembly of (densely labelled) barcodes using a matrix profile approach.

The experimental barcodes are generated through a multi-step process. Detailed experimental procedures are provided in S1.1–S1.5 in S1 Text. In brief, bacterial DNA is extracted, placed in agarose plugs to reduce fragmentation, labeled using a competitive binding reaction with YOYO-1 and netropsin, and visualized in nanofluidic devices using fluorescence microscopy. Some of the experiments have DNA molecules that are not in their equilibrium extension during imaging, i.e., they are decreasing in length (shrinking) over time. To remove such molecules from further analysis, the experimental datasets are subject to an automated shrink-filtering method (See S1.6 in S1 Text). In the final step, kymographs (movies) of non-shrinking DNA molecules are converted to experimental barcodes. A kymograph is composed of one-dimensional traces of the molecule over time. An experimental barcode is a one-dimensional data series of aligned and time-averaged kymograph.

**Fig 1:**
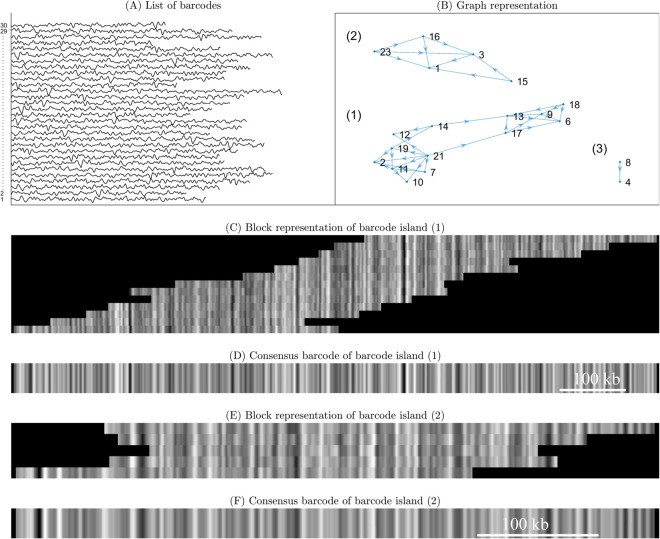
Schematics of de novo barcode assembly. As an illustration, we here show the expected output of the *de novo* OGM assembly for a list of 30 barcodes. Each barcode corresponds to the intensity at different positions along a fluorescently-stained single DNA molecule. (A) A list of stacked input barcodes *b*_*i*_, where *i* is the index of the barcode. The barcodes are z-normalized (mean = 0 and standard deviation = 1) and intensities are shifted for visualization. (B) Graph of barcode islands, vertices representing barcodes and edges representing overlaps between pairs of barcodes. (C) Block representation for barcode island (1), representing a partial solution to the *de novo* OGM assembly. Along the y-axis, we have barcodes (vertices) from barcode island (1) in panel B. Along the x-axis, we have positions along a DNA molecule, and the pixel intensity represents signal strength at those positions. (D) Consensus barcode for barcode island (1). (E) Block representation for barcode island (2). (F) Consensus barcode for barcode island (2). Barcode Island 3, which contains only two barcodes, is not shown.

The procedure for generating synthetic barcodes, which can be seen as a one-dimensional data series (Fig 1A), is given in S1.7 in S1 Text.

With synthetic and experimental barcodes at hand, the *de novo* OGM assembly problem and our solution is conceptually visualized in Fig 1. Barcodes have to be grouped into “barcode islands” without the help of a reference genome (based on pair-wise similarity). A barcode island is composed of barcodes where each barcode has (significant) overlap with at least one other barcode in that island. The barcode islands can be represented in three ways: (i) through a table, (ii) through a graph representation (Fig 1B), or (iii) through a block representation (Fig 1C, 1E shows barcode islands 1 and 2). When the coverage is high enough, the number of barcode islands will mirror the number of chromosomes in a genome. Finally, each barcode island may be averaged to get a consensus barcode representation. A consensus barcode (Fig 1D, 1F) for a barcode island is an analogue of a contig in the DNA sequence assembly community, i.e., it is an (amplitude-adjusted) average of the barcodes in the barcode island.

### Overview of the DOGMA pipeline

**Fig 2:**
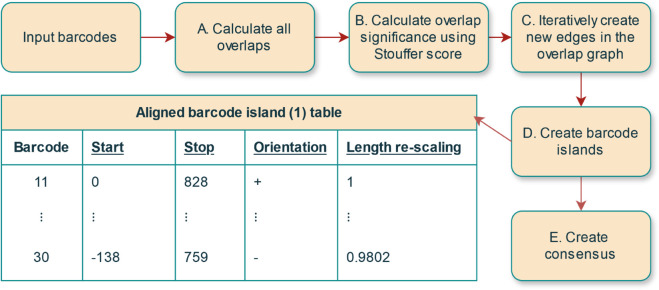
DOGMA pipeline. The first part of the full pipeline consists of the OGM assembly pipeline with 4 steps: A) Calculate overlap scores between all pairs of input barcodes. B) Calculate Stouffer scores between all pairs of input barcodes. The Stouffer score is "length-insensitive", in the sense that it measures the significance of the overlap as compared to a null model for the given overlap length. Making use of length-insensitive scores is needed since lengths of the overlaps found in the first step varies. C) Iteratively create new edges in the barcode islands graph passing the criteria for adding a new edge. D) Create barcode islands. This includes creating aligned barcode island tables for each barcode island. An output table for each of the barcode islands contains start position, stop position, orientation (’+’ for forward and ’-’ for reverse) and length re-scaling factor (factor by which a barcode needs to be re-scaled in order to be matched to the other barcodes) for each of the barcodes in the barcode island. Finally, E) in the optional consensus pipeline a consensus barcode is generated based on the output from the assembly pipeline. This includes amplitude and iterative correction of the barcode islands. The default parameter values are listed in S3 Table in S1 Text.

We use an overlap-layout-consensus approach [13] for the *de novo* OGM assembly (see Fig 1) of the densely labelled DNA barcodes. The *DOGMA* method consists of two consecutive pipelines. First, in the assembly pipeline (illustrated in Fig 2A–2D), we make pairwise comparisons between all pairs of barcodes, using a Matrix Profile (MP) based approach, and then pairs of barcodes are iteratively merged into an overlap graph if they satisfy consistency conditions (to be defined below). Following the assembly pipeline, there is an optional step, the consensus pipeline, for generating a consensus barcode (see Fig 2E). We provide a list of clarifying remarks after explaining the main steps of the OGM assembly pipeline.

#### OGM assembly pipeline, details.

1. In the first step of the method, we pairwise compare all of the barcodes *b*_*i*_, i=1…N, where *N* is the number of barcodes. Each pair consists of a *reference* and a *query* barcode. The *query* is fixed, but the *reference* is allowed to be length-rescaled and its direction can be reversed. For each *reference-query* barcode pair *i*,*j* we then find the optimal local overlap score *C*_*i*,*j*_ with pre-selected overlap window width *w* using MP to optimize the overlap position *l*, length re-scaling factor *r* and the orientation direction *d*, i.e.
  $$C_{i,j}=\max_{l,r,d}\frac{QT(l,r,d)-w\cdot \mu_{i}(l,r,d)\mu_{j}(l,r,d)}{w\cdot \sigma_{i}(l,r,d)\sigma_{j}(l,r,d)}$$ (1)
  where *QT* is the corresponding dot product at positions $l=\{l_{1},l_{2}\}$ for the re-scaled and oriented *reference i*. In Eq 1, $\mu_{i},\mu_{j}$ are the means and $\sigma_{i},\sigma_{j}$ are the corresponding standard deviations. We here also calculate the left-over overlap score $C_{i,j}^{\text{leftover}}$ by extending the overlap, i.e. we calculate the score over the overlapping positions between *reference* and *query* that were *not* included in the local overlap. See S2.1 in S1 Text for implementation details of the MP based scores.
2. The significance of the optimal overlap scores between any *reference* and *query* barcode is calculated using a null model [23]. The scores *C*_*i*,*j*_ and $C_{i,j}^{\text{leftover}}$ are first converted to p-values $p_{\text{local}}$ and $p_{\text{leftover}}$. Only overlap pairs that pass the p-value thresholds for local and leftover overlaps are considered significant, i.e. $p_{\text{local}}<p_{\text{local}}^{\text{thresh}}$ and $p_{\text{leftover}}<p_{\text{leftover}}^{\text{thresh}}$. The significant overlap p-values are finally converted to a combined score $s_{\text{Stouffer}}$ using Stouffer’s method [24],
  $$s_{\text{Stouffer}}=\frac{\Phi^{-1}(1-p_{\text{local}})+\Phi^{-1}(1-p_{\text{leftover}})}{\sqrt{2}}$$ (2)
  where *Φ* is the standard normal cumulative distribution function. See S2.1 in S1 Text for further details on parameters used in the null model.
3. Significant barcode pairs are then iteratively merged into a *graph G*, starting from the highest $s_{\text{Stouffer}}$ score. Vertices of the graph are the barcode indexes and edges are the overlaps. At each step the indices of the new pair of *query* and *reference* (corresponding to the next highest score) are compared to existing vertices in the *graph* to check if it is *consistent*. If the positions are within the allowed pixel difference *pxDiffSetting* and the length-rescaling factor is within the allowed scaling factor *scDiffSetting* (S3 Table in S1 Text provides the default values), then we consider the pair of barcodes *consistent*. This then provides us with four cases (schematics of how this works in practice are shown in S14 Fig in S1 Text):
  - Both indices are new. This is the simplest case, and we create a new component in the graph containing *query* and *reference*. At the same time we initialize a *merge* table, containing vertices of barcodes pairs (initialized to be empty).
  - The *query* index is new. We first check if *query* already exists in the *merge* table of the graph component that the *reference* is a member of. If it does, and the two pairs are *consistent*, we add *query* and the new edge to the graph component. Otherwise we update the *merge* table with a new *query*, *reference* pair.
  - The *reference* index is new. We first check if *reference* already exists in the *merge* table of the graph component that the *query* is a member of. If it does, and the two pairs are consistent, we add *reference* and the new edge to the *query* graph component. Otherwise we update the *merge* table with a new *query*, *reference* pair.
  - Both indices already exist. In this case we check if *merge* tables in the *reference* and *query* graph components already have a different pair of barcodes between these two components. If so, we check if the two pairs are consistent. Otherwise we update the *merge* tables with a new *query*, *reference* pairs.
4. Block representations of barcode islands are generated in the form of tables (See table Fig 2 for an example) from the graph representation with one barcode island per graph component.

A few clarifying remarks:

(i) The MP-based approach is used to find overlapping features of the same length among the barcodes. This is important as the lengths of the barcodes themselves and their overlaps are of varying length. The overlap window width (which is used as a parameter for the MP algorithm) is here chosen to be 150 kilobasepairs, which for the experimental set up in this paper was equal to approximately *w* = 300 pixels. This choice is motivated based on the results in a previous study [9]. S5 Fig in S1 Text gives additional motivation why this is a good choice for the window width.

(ii) *C*_*i*,*j*_ is the largest score among the sub-barcodes (each of length *w*) of an overlap between two barcodes. The leftover overlap score $C_{i,j}^{\text{leftover}}$ is the Pearson correlation coefficient (PCC) for the "remaining" overlap pixels, which are not covered by the overlap. Leftover scores are used to better control that the position we found with the local alignment is better than "random". $s_{\text{Stouffer}}$ score is only calculated for significant overlaps and defines the order in which barcodes are added to the overlap graph.

(iii) We check for consistency while creating the overlap graph in order to remove most of the remaining false positives among the significant barcode pairs.

(iv) We use $p_{\text{local}}^{\text{thresh}}=p_{\text{leftover}}^{\text{thresh}}=0.05$, meaning that we allow for 5% percent of false positives. Note that some overlaps are removed using the consistency checks, meaning the final false positive rate is in general slightly lower.

#### Pipeline for generating consensus.

As an additional step after generating the barcode islands, the barcode islands can be converted into consensus barcodes (See S4 in S1 Text). In our approach there are two sub-steps where the barcode island are (1) amplitude adjusted and (2) re-aligned:

1. In order to amplitude adjust the barcodes in an island, we first calculate pairwise PCC scores (where the number of overlapping pixels is no less than *w*) for all barcodes in the barcode island. We then cluster the barcodes using single-linkage clustering (matlab’s linkage) into a hierarchical tree with a cut-off threshold score of 0.5 (implemented using matlab’s cluster with a cutoff parameter). We finally adjust the amplitudes of the barcodes in the barcode island by going through the nodes of the hierarchical tree iteratively, starting with highest scoring pair of barcodes *i*_1_ and *i*_2_ (the pair which have the highest PCC score in their barcode island). We start by setting the mean $\tilde{\mu }$ to be $\tilde{\mu }=\frac{\mu_{i_{1}}+\mu_{i_{2}}}{2}$, and $\tilde{s}$ the square root of the mean of the variances $\tilde{s}=\sqrt{\frac{\sigma_{i_{1}}^{2}+\sigma_{i_{2}}^{2}}{2}}$. We then apply the correction
  $$b_{i}(x)\to \left(b_{i}(x)-\mu_{i}\right)\frac{\tilde{s}}{\sigma_{i}}+\tilde{\mu },$$ (3)
  to both *i* = *i*_1_ and *i* = *i*_2_. After adjusting the amplitudes for the best pair, we continue through hierarchical clustering tree, by adjusting the pair of barcodes with the next best PCC score, until we have gone through all the pairs.
2. The draft consensus barcode is calculated as a mean over the rows of block representation of amplitude adjusted barcode island (zeros are not counted). The barcodes of the barcode island are then re-aligned individually along the consensus barcode by adjusting the length within a maximum allowed stretch/compression of 5% . This creates a new block representation of re-adjusted barcode island. We then iterate steps 1-2 of the consensus pipeline X times (here *X* = 5). This creates an amplitude re-adjusted block representation of the barcode island.

Further clarifying remarks:

(i) The barcode islands are amplitude adjusted in order to correct for differences in overall photon counts of individual DNA barcodes due to, for instance, photobleaching effects.

(ii) The barcode islands are re-aligned on the new consensus in order to correct for size and scaling differences in the original alignment due to, for instance, limited range of length re-scaling factors used in the first step of the main algorithm.

(iii) The amplitude correction formula used in step 1 makes sure that two adjusted barcodes *b*_1_ and *b*_2_ have the same mean $\tilde{\mu }$ and standard deviation $\tilde{s}$ for the overlapping region.

## Results

In this Results section we first consider a synthetic dataset with varying signal-to-noise ratio and then a real experimental dataset (see Table 1). Both of these have a known genomic sequence that can be used to validate the results. The synthetic datasets are generated from an *Escherichia coli* strain (sequence ID NC_000913.3 ), while the experimental dataset is from a different *Escherichia coli* strain (sequence ID CP023853.1). The average barcode length for the synthetic dataset is 850 pixels, while for the experimental dataset the estimated average length is 855 pixels, which corresponds to roughly 400 kilobasepairs.

**Table 1:** Experimental data for the *de novo* OGM assembly.

Sample	#Experiments	#Filtered	Reference length
Synthetic NC_000913.3	200	-	12659 px
Experimental CP023853.1	928	752	11760 px

Some of the experiments had molecules which decrease in length (shrink) over the time. To remove such molecules, experimental datasets were first run through a shrink-filtered step (See S1.6 in S1 Text) before running the DOGMA pipeline.

### Synthetic data

**Fig 3:**
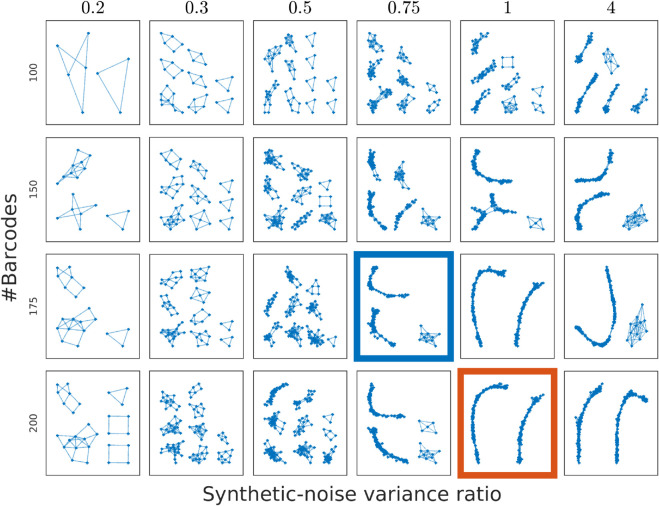
Graphs of barcode islands for different synthetic-noise variance ratio and number of synthetic barcodes. The number of barcodes is varied between 100 and 200, while synthetic-noise variance ratio is varied between 0.2 and 4. Note that for larger synthetic-noise variance ratio and number of barcodes we obtain two barcode islands as an output. Having two barcode islands is equivalent to having a complete genome as they represent two parts of a circular genome. The example in the red square is analyzed in Fig 4, while the one in the blue square is in S17 Fig in S1 Text. Parameters used to generate the example are provided in S2 Table in S1 Text.

We begin the analysis with a synthetic dataset (’Synthetic’ in Table 1). This sample contains 10 datasets with varying synthetic-noise variance ratios (SNVR). The definition of SNVR is given in S1.7 in S1 Text. Since for the synthetic barcodes we can vary the SNVR, in Fig 3 we show how the number and size of the barcode islands depends on this quantity. It becomes clear, as expected, that low SNVR and small number of barcodes makes it difficult to find any barcode islands, while a higher SNVR and a larger number of barcodes has a higher potential to lead to formation of a single barcode island.

**Fig 4:**
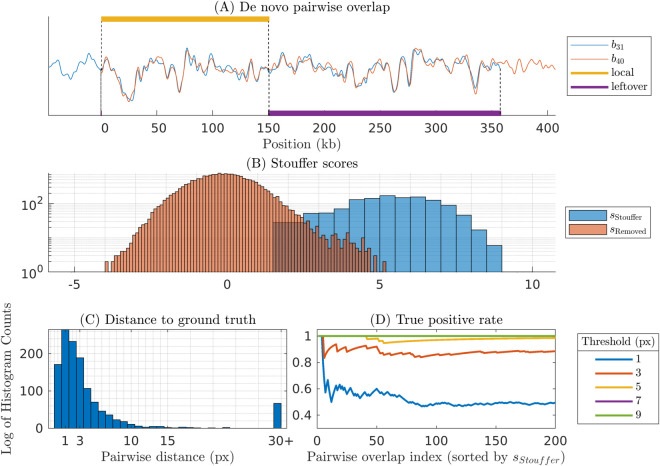
Pairwise Stouffer scores and distance validation. (A) Our method starts by computing two scores *C* and $C^{\text{leftover}}$ between barcode pairs. (B) Both these scores are converted to p-values, and the scores failing tests based on $p_{\text{local}}^{\text{thresh}}$ or $p_{\text{leftover}}^{\text{thresh}}$ are discarded. The remaining scores are combined using Stouffer score $s_{\text{Stouffer}}$. For comparison, we also calculate the Stouffer scores, $s_{\text{Removed}}$ for those barcode pairs which were discarded. (C) The distance to ground truth (GT) histogram for significant barcode pairs visualizes false positives. Note that around 5% of the barcode pairs have distance larger than 30 pixels, corresponding to our p-value thresholds of 0.05. (D) True positive rate at different distance thresholds as compared to the ground truth positions, with the pairwise overlap index sorted by $s_{\text{Stouffer}}$. This plot shows that we often can get small positional variations as compared to the ground truth, but we neglect these since they appear mainly because of limited number of length re-scaling factors used. A plot showing reference based positions for barcodes in (A) is given in S6 Fig in S1 Text.

Fig 4 illustrates and validates the pairwise comparison part of the DOGMA algorithm (step 1 and 2 in the OGM assembly pipeline in Materials and Methods). First, an example of *de novo* pairwise overlap for synthetic-noise variance ratio  = 1, #barcodes=175 case is shown in Fig 4A. In Fig 4B we show two histograms of scores: Stouffer scores, $s_{\text{Stouffer}}$, and scores for barcode pairs that do not pass the thresholds, ($s_{\text{Removed}}$). Note that these two histograms have an overlap since it is enough for an overlap to fail one of the p-value thresholds in order to be discarded. This motivates our use of two p-values - if we had only used a single score, we would have had a larger number of false positives. For validation of the pairwise comparison for synthetic data results we use the ground truth (GT) positions of the experimental barcodes along a theoretical reference barcode. The theoretical reference barcode (hereafter called theoretical barcode) refers to the expected barcode made by simulating the intensity profile of the reference genome. Since both the experimental barcodes and the theoretical barcode here are synthetic, we know that the distance between starting positions of two barcodes along the theoretical barcode should match closely to the distance between starting positions of the two barcodes when matching them to each other *de novo*. In Fig 4C we show the pairwise distances to the ground truth for the scores that pass the thresholds. An example of how these distances are calculated is given in S2.3 in S1 Text. For this dataset the number of false positives (overlaps that were at least 40 pixels away from the reference based overlap) is 5.29%, falling close to the 5% p-value threshold. Fig 4D shows the true positive rate, i.e. percentage of the scores corresponding to pairwise distance less than the distance threshold. The corresponding GT positions are given in S6 Fig in S1 Text. The mean distance to the GT of true positives in the histogram in Fig 4C is 2.9 pixels, and standard deviation is 3.0 pixels.

**Fig 5:**
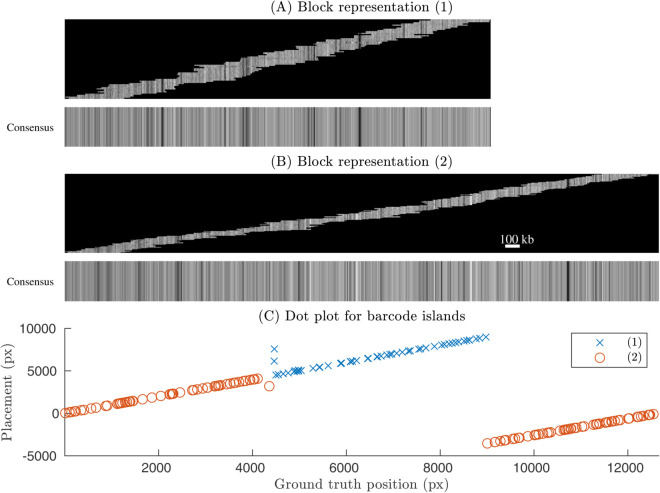
Barcode islands and corresponding dot plot validation for synthetic barcodes. (A-B) Block representation for barcode islands (1-2) along with the associated consensus barcodes. (C) Corresponding dot plot. The x-axis is the starting position along the theoretical barcode, and the y-axis is the starting position along the barcode island after alignment to the theoretical barcode. Note that the two islands cover two distinct (but possibly overlapping) regions along the chromosome. In this example the dataset with synthetic-noise variance ratio  = 1 and # barcode = 200 is considered (see Fig 3). See S12 Fig in S1 Text for the zoom-in of the overlap between two barcode islands. S17 Fig in S1 Text show another example with three barcode islands).

Next we seek to validate the full *de novo* OGM assembly results, i.e. quantify the accuracy of the barcode islands (see step 3 in the OGM assembly pipeline in Materials and Methods). To this end, we first find a position for the full barcode island along the theoretical barcode. In order for this procedure to work also for experimental data we first align all the barcodes from the barcode island against the ground truth reference barcode to get a ground truth alignment table (for the alignment length here we use the length of the barcode). These scores are then converted to p-values (see S2.3 in S1 Text for details). The lowest p-value barcode is set as an anchor barcode. The table of the barcode island is then matched to the ground-truth alignment table as in S3.1 in S1 Text. We show the result of this validation in Fig 5. Fig 5A–5C shows the block representation of the most populated barcode islands for the example considered. Fig 5D–5F shows dot plots for the distances to the ground truth for each of the barcode islands. The distances are sorted based on their position on the genomic reference, showing that the three islands represent three unique regions along the genome. The average distances to the GT for true positives for each of the barcode islands were 3.5 and 11.9 pixels, and std. dev 2.8 and 9.8 pixels. The corresponding percentages of false positives were 6.7%. Note that here we use 30 pixels as maximum variation from the GT, as we get extra positional variation from anchoring the whole barcode island as opposed to individual barcodes. Corresponding maps of matching individual barcodes of each barcode island to the theoretical reference barcode are shown in S15–S16 Fig in S1 Text.

### Experimental

**Fig 6:**
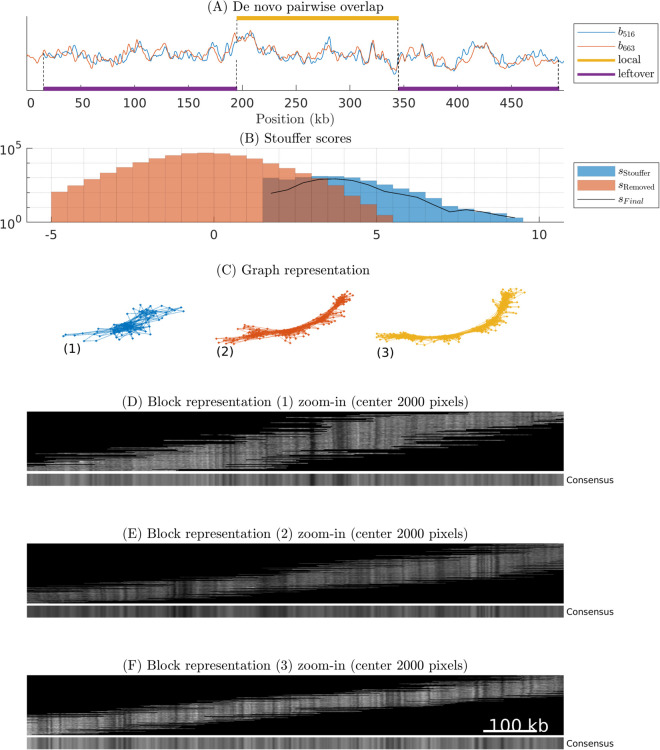
Pairwise overlap and barcode islands for the experimental genome. (A) Overlap between two experimental barcodes and (B) Stouffer scores histogram. We here additionally show $s_{\text{Final}}$ scores which is a subset of Stouffer scores that discard the pairs removed while creating barcode islands. (C) Graph representation of barcode islands. (D-E) Block representation of barcode islands and associated consensus barcode island. Full block representations are given in S7–S9 Figs in S1 Text.

Having validated our procedure and choice of parameters using synthetic barcodes, we now turn to OGM assembly of experimental DNA barcodes. The main result here is that we are able to assemble the barcodes into three barcode islands, with the number of experimental barcodes at hand (see Table 1). An example of *de novo* pairwise overlap is shown in Fig 6(A). Note the visual similarity between the two barcodes (both for local and left-over overlap). In Fig 6(B) we show three histograms: Stouffer scores $s_{\text{Stouffer}}$, scores for barcode pairs that do not pass the thresholds ($s_{\text{Removed}}$), and histogram of the scores for all overlaps kept in the barcode islands, $s_{\text{Final}}$ (this is a subset of the Stouffer scores). Note that the peaks of the Stouffer scores and discarded scores are well separated. The histograms overlap because it is enough to fail one of the two p-value thresholds in order for the score to be removed. In Fig 6(C) we show the graph representation of the barcode islands, and in Fig 6(D)–6(F) we show block representations for the center 2000 pixels for barcode islands after the iterative amplitude rescaling procedure.

**Fig 7:**
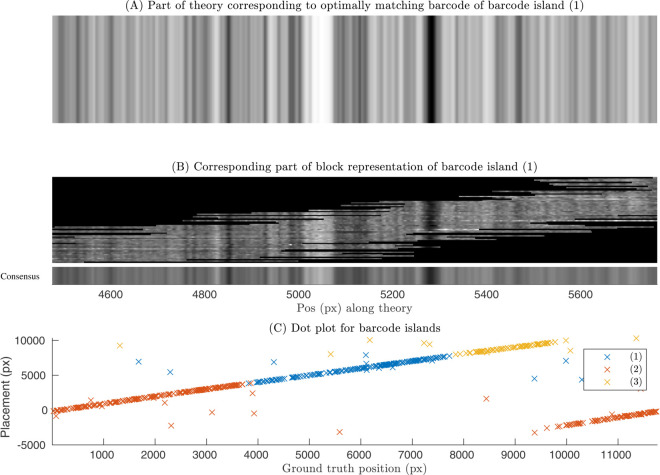
Indirect validation for the experimental sample. We calculate the theoretical DNA barcode for the experimental sample, using the known DNA sequence as input [23]. We then match all experimental individual barcodes for the experimental sample against the theoretical barcode. We finally identify the part of the theoretical barcode corresponding to each barcode island. (A) Theoretical barcode associated with barcode island (1). (B) Block representation of barcode island (1). (C) Dot plot, which shows how the placement positions of the individual barcodes within each of the three barcode islands corresponds to the indirectly predicted ground truth positions. The x-axis is the starting position along the theoretical barcode, and the y-axis is the starting position along the barcode island after alignment to the theoretical barcode. Here as the indirect ground truth we take the optimal placement position of the individual barcode along the theoretical barcode. Some of the obvious false positives would be further removed if we removed the worst matching barcodes by using p-value thresholding for the placement along the theoretical barcode. See S13 Fig in S1 Text for the visualization of the overlap between barcode islands (1) and (2). S11 Fig in S1 Text shows visualization between the consensus and the theory barcode for the selected region.

Validating the OGM assembly of experimental barcodes is more challenging than for synthetic barcodes where the ground truth is available. We therefore use an "indirect" validation strategy using theoretical barcodes based on the complete genome sequence. Experimental barcodes are aligned to the reference genome and the alignment positions used to create an indirect ground truth table in the same way as for synthetic experiments. Fig 7(A) shows a block representation of the region of the theoretical barcode anchored to the highest scoring individual barcode alignment positions. Fig 7(B) shows the corresponding part of the barcode island. Fig 7(C) shows the dot plot for the distances to the ground truth for both barcode islands. Note that together the three barcode islands cover the whole genome. A number of false positives (barcodes not falling on a straight line) in these plots come from either individual barcodes matching to the wrong part of the theoretical barcode or from individual barcodes wrongly included in the barcode island.

## Discussion

Below we make some technical comments about our OGM assembly algorithm and briefly discuss how in the future one may improve, build on, or use the DOGMA pipeline.

The largest computational cost of our method comes from calculating all-to-all pairwise overlaps between individual barcodes. The scaling of computational time grows quadratically with the number of barcodes. Typical running time for the full pipeline in Fig 6 is < 10 minutes on a desktop computer with 32 cores. This makes DOGMA feasible for bacterial-sized genomes, and highlights its scalability for small to moderate barcode sets To deal with larger chromosomes and larger sets of barcodes, one could potentially introduce an indexing or downsampling scheme for speeding up the identification of barcode pairs with significant overlaps.

The strength of using p-values in our calculations is that we have control of the expected number of false positives. Moreover, with heuristic scores it is not obvious how to compare alignments of different lengths, which is automatically solved by the use of p-values. In the study we did not quantitatively analyse the false negatives, i.e. "correct" barcodes pairs which are wrongly discarded in the OGM assembly pipeline. The main effect of such false negatives is that we need more barcodes to achieve complete OGM assembly. Thus, false negatives are less detrimental to the assembly than false positives, the latter which potentially can lead to "nonsensical" barcodes islands if not removed from the analysis.

As a final step, after calculating the barcode islands, we generate a consensus barcode as an optional step (which serves as a single representative of the barcode island). The consensus barcode can be used in further downstream analysis tasks. For bacterial species identification, the barcode islands can be used as is or, alternatively, the barcode island can be oriented on a theoretical barcode using one or a few "best" representatives of the barcode island. Which approach is best suited for species identification purposes is left for future studies.

One challenge when creating the consensus barcode from the assembly is that different molecules typically have overall differences in intensity which may originate from different illumination light intensities, photobleaching, or slight differences in the staining. In our consensus method, we correct for this by adjusting the amplitudes of individual barcodes before merging them into a consensus barcode. This adjustment provide robustness to our consensus method, but through the adjustment step we loose some information about overall barcode intensity.

In our consensus approach, we also included a realignment step. After creating a barcode island and making a consensus out of the individual barcodes contained in the island, we align the individual barcodes on its consensus. Then we use the alignment positions to create a new barcode island. This step is computationally fast as we need to compare the individual barcodes only against its consensus, rather than all individual pairwise alignments. The re-aligned barcode islands can be used to investigate if we have included some barcodes which are less certain to be in the final alignment, or if we erroneously merged two large groups of barcodes. We leave this investigation for future studies.

The main novelty in DOGMA is its ability to perform de novo assembly, and generate consensus barcodes, from densely-labelled barcodes. In this study we used barcodes labelled using the competitive-binding assay, but we point out that our method should work, after suitable adaption, also for other types of dense-labelling methods such as dense enzymatic labeling [14] or DNA melting-based labeling [19]. Moreover, by utilizing the combined p-value approach from [24], DOGMA could potentially also be used for assembling barcodes from DNA molecules with multiple label types.

## Conclusions

In conclusion, we have presented a comprehensive method for the *de novo* OGM assembly of densely labelled DNA barcodes. By calculating optimal overlap scores, establishing significance thresholds, and iteratively merging significant barcode pairs, our method effectively aligns barcodes and validates their positions. The validation of assembly for a set of experimental barcodes against a theoretical reference genome demonstrates the accuracy and reliability of our approach. *DOGMA* contributes to the advancement of *de novo* OGM assembly techniques and opens avenues for further research in genomics and DNA analysis.

Through the use of our *de novo* OGM assembly (barcode islands or consensus barcodes) one can for instance identify structural variations or aid the DNA sequence assembly of complex genomic regions. It can also be used in microbial and metagenomic studies by assembling and characterizing genomes from mixed samples and in detecting epigenetic modifications such as DNA methylation and hydroxymethylation [5,6]. The backbone of the DNA is labelled in any case, and the ability to extract additional information from this channel is potentially of use.

Looking forward, the integration of OGM *de novo* assembly with other sequencing technologies has the potential of enhancing our understanding of genomic structure and function. We hope that this study, along with its publicly available software, will pave the way for use of barcode islands and consensus barcodes in genomic applications, potentially finding uses in personalized medicine, evolutionary biology and the study of genetic diseases.

## Supporting information

S1 Text Supplementary methods. Contains additional descriptions and supporting figures for the DOGMA pipeline.
(PDF)
