# Supplementary material for: DOGMA: de novo assembly of densely labelled optical DNA maps using a matrix profile approach
Source: PLoS One. 2025 Dec 1;20(12):e0335633. doi: 10.1371/journal.pone.0335633 (PMC12668544; doi:10.1371/journal.pone.0335633)
Supplement: S1 Text — Contains additional descriptions and supporting figures for the DOGMA pipeline. (PDF) [file pone.0335633.s001.pdf]

Supplementary Materials for "DOGMA: *de novo* assembly of densely labelled optical DNA maps using a matrix profile approach"

S1 Experimental details

S1.1 Bacterial samples

Bacterial strains were grown in Luria-Bertani (LB) broth, with 1,5% agar for solid medium, and stored at -80°C in 10% DMSO stocks.

S1.2 DNA extraction

The protocol for extracting DNA has been described previously [1]. Briefly, bacterial cultures were grown overnight, 250 μL of culture was pelleted and the bacteria were enclosed in an agarose plug (100 μL). To protect the DNA from fragmenting, the entire lysis protocol was carried out inside the agarose plug. The bacteria were lysed, treated with RNase A and proteinase K, and finally washed in TE-buffer. These agarose plugs were then stored at 4°C until their intended use. The agarose plugs were melted at 70°C for 10 minutes and subsequently conditioned at 42°C for another 10 minutes. Following conditioning, the plugs were then digested with 1 U of Agarase (Thermo Fisher Scientific) at 42°C for 1 hour. DNA concentration was then determined by using Qubit dsDNA Broad Range Assay kit (Invitrogen).

S1.3 DNA labelling

Densely labelled DNA was obtained by a single-step competitive binding (CB) based staining reaction, as described previously [2]. The CB staining reactions were performed by adding 4.8-6.8 μM DNA, 0.48-0.68 μM YOYO-1, and 144-204 μM netropsin in a 10:1:300 ratio to a 0.5X TBE (Tris-Borate EDTA) 10 μL solution. DNA from bacteriophage λ was added as a size reference (48,502bp) to make up to 30% of the DNA of the staining reactions. Finally, an incubation of 50°C for 30 minutes was followed by a 10-fold dilution with MQ-water and 2μL of β-mercaptoethanol (BME).

S1.4 Data acquisition

DNA molecules were stretched for imaging purposes using a nanofluidic device whose fabrication is described elsewhere [3]. These devices are designed to have two sets of two loading wells, each set connected through a separate microchannel. Additionally, both microchannels connecting the loading wells are connected to each other by 120 parallel nanochannels, each measuring 100x150 nm^2 in dimension and extending 500 nm in length. To ensure uniform conditions, the chips underwent pre-conditioning with 0.05x TBE buffer supplemented with 2% BME v/v prior to sample loading. Subsequently, 10μ L of diluted staining reaction (sample) was introduced into one of the loading

wells, while the others contained the same buffer used for pre-conditioning. DNA loading into the nanochannels was facilitated through pressure-driven N_2 flow.

The imaging procedures employed an inverted fluorescence microscope (Zeiss AxioObserver.Z1) equipped with alpha Plan-Apochromat 100x/1.46 Oil DIC M27 objective (420792-9800, Zeiss), Colibri 7 light source (Zeiss), and a Photometrics Prime 95B 22mm Scientific CMOS (sCMOS) camera. Systematic acquisition of videos of up to 100 frames was carried out for each molecule, utilizing an exposure time of 100 ms and a FITC filter set (Zeiss).

S1.5 Processing of experimental data

Barcodes were generated from movies using a previously developed custom MATLAB software called 'lldev' [4] [5]. In short, DNA molecules are detected in each time frame within a movie. The detected DNA molecules were then turned into kymographs, where each row of pixels represents the intensity along the DNA molecule in a single time frame. The kymographs are aligned to compensate for the small thermal fluctuations of the DNA inside the nanochannels. Next, the aligned kymographs are time-averaged, rendering a 1D intensity trace along the extension of the DNA molecule, referred to as a barcode.

Since the confinement of DNA within nanochannels does not immobilize the DNA, a relaxation time is required for the DNA to attain equilibrium in its final extension within the nanochannel. The relaxation time of DNA molecules confined within nanochannels has been observed to scale with the square of the DNA contour length [7]. Consequently, longer DNA strands require longer relaxation time compared to shorter ones. Therefore, upon capture, long DNA molecules exhibit a dynamic behavior characterized by a transient "shrinking" state, indicating that the molecules have not yet attained their relaxed state or equilibrium size within the nanochannel. Kymographs of DNA molecules which were still in a shrinking state yield barcodes with features that may obscure each other, thereby compromising their accuracy.

To ensure the accuracy of our barcodes by mitigating the inclusion of shrinking kymographs in our analysis pipeline, we developed a shrink-filtering algorithm to identify and remove kymographs of DNA molecules that were still in a shrinking state. Our algorithm is used before the kymograph alignment to improve the integrity of subsequent data analysis by systematically identifying and removing such kymographs.

An important part of the shrink-filtering algorithm is based on the detection of features along the DNA molecule. In this context, a feature is any noticeable intensity peak or valley along the DNA molecule that is consistent across all the time frames of a kymograph and that are detectable by the NRAlign function within the lldev software [6].

To identify shrinking kymographs, each DNA molecule is divided into left and right halves. This is done by using NRAlign to identify prominent features on the leftmost, middle, and rightmost sections of the kymograph. The $around_feat$ parameter defines the fraction of features to be included for each section. Outlier features are identified and excluded using the Matlab *dbscan* function, while the remaining features are averaged to create representative features for the left, middle, and right sections. The left half is defined as the region between the leftmost and middle representative features, while the right half spans from the middle to the rightmost feature.

The rate of shrinking for each side is determined by fitting a first-order polynomial to the size of the molecule (in pixels) over time (100ms per frame). The side with the most negative slope is selected, and its size data is input into Matlab's findchangepts function with parameters shown in Table 1.

The function identifies changepoints in molecule size over time, generating a stability plot. A kymograph is classified as "shrinking" if the stability plot shows more than one section (i.e., at least one changepoint) or if the shrinking rate exceeds 2px per 100ms. These criteria align with our kymograph alignment algorithm's ability to perform local stretching. If a kymograph is flagged as shrinking, the algorithm searches for a stable region of at least 10 frames (1 second) with no changepoints. This "latest stable region" must occur near the end of the kymograph (tuned by the *restrict* parameter). If the shrinking rate in the stable region is below 2px per 100ms, the earlier shrinking section is cropped, and the kymograph is considered "fixed." Kymographs without a stable region are

Table 1: Parameters used for the shrink-filtering algorithm

Name	Description	Value
$around\_feat$	fraction of features used for representative feature	0.10
$restrict$	fraction of time elapsed to classify latest stable region	0.9
shrink_threshold	Minimum improvement in total residual error of mean for each changepoint (findchangepts)	40

Table 2: Parameters used for synthetic experiments

Name	Description	Value
N	number of random fragments	200
pxpsf	Point spread function (px)	2.72
σ_L	Standard deviation for fragment length	100
snvr	Synthetic-noise variance ratio	1:1:10
isC	Whether experiment is circular	1
σ_{theory}	std for theory	20 (image counts)
m_{noise}	Additive mean noise	200 (image counts)
$\sigma_{re-scaling}$	std for length re-scaling	0.05
m_L	mean fragment length	850

filtered out.

Only non-shrinking or fixed kymographs proceed to the alignment and barcode generation stages, ensuring that our barcodes reflect DNA molecules in their relaxed state, improving the accuracy and reliability of the analysis.

S1.6 Generating synthetic barcodes

To get a realistic looking synthetic barcode all we need is (i) theoretical YOYO-1 binding probability map for a DNA sequence [8] (ii) a method for noisifying such probability maps, and including effects due to the imaging system's point spread function (PSF).

The first part is to generate the theoretical barcode, which is done using our previously developed methodology [8], with the PSF convolution disabled. The theoretical barcode is then kept fixed.

The noise part is then generated so that two overlapping regions between theoretical barcode and synthetic barcode will not be identical. To generate the noise part, we first z-normalize the theoretical barcode, scale it by σ_{theory} and add the additive mean noise m_{noise}. We then randomly cut out a fragment with mean length m_L and re-scale it using a random number generated from normal distribution centered at 1 and with standard deviation $\sigma_{re-scaling}$. Additive noise is generated for each fragment as a normally distributed vector with a standard deviation

$$\sigma_N = \sqrt{\frac{\sigma_{theory}^2}{snvr}} \tag{1}$$

Finally, we convolve with a Gaussian with sigma of $pxpsf$ in order to mimic the experimental conditions. $pxpsf = 2.72$ corresponds to 110 nm/px and DNA stretching to 0.3 nm/bp. All the parameters used to generate the synthetic experiments are given in Table 2. Examples of synthetic barcodes are given in the Main text, Fig 1.

S2 Definitions and parameters used in DOGMA

S2.1 Local and leftover overlap scores

Both local and leftover overlap scores are based on calculating the Pearson Cross Correlation (PCC) coefficient. The computational approach to calculate these scores differs slightly. To calculate local overlap scores, we need to run through a large number of placement attempts. For leftover overlap score, there is only a single placement. We present algorithms used for most methods in detail below.

Local overlap scores are calculated using matrix profile (MP) algorithm [9] using the command
scamp –window=300 –input_ a_file_ name=name1 –input_ b_file_ name=name2 –num_ cpu_ workers=30

Here *window* is the length of the local overlap (w in the main text), input $name1$ is a concatenated query barcode, together with its reverse complement, length re-scaled to different lengths using length re-scaling factors of 10% with step size of 1% (see Table 3), and $name2$ is all the reference barcodes (except the query barcode) concatenated together. The associated algorithm used in the DOGMA pipeline is found in Algorithm 1.

Given two barcodes $b_1 = \{b_1(1), \ldots, b_1(k_1)\}$ and $b_2 = \{b_2(1), \ldots, b_2(k_2)\}$, the local alignment algorithm calculates a matrix profile P_{b_1,b_2} and a matrix profile index T_{b_1,b_2}. In our case, each of the scores P_{b_1,b_2} and corresponding matrix profile index value T_{b_1,b_2} can be described by position of first overlap pixel on b_1, position of first overlap pixel on b_2, orientation of barcode b_1, and length re-scaling factor of barcode b_1, thus giving full information about the overlap between barcodes. Since we are usually interested only in the "best overlap", we calculate the four parameters: p_A - location along barcode b_1, p_B - location along barcode b_2, p_O - orientation for barcode b_1, p_S - length re-scaling for barcode b_1) only for the best position (See Algorithm 2).

function : calc_overlap_mp
Input : $barStruct$, w, sF
Output : oS

```
for k ← 1 to N do
    [namesBar, stridx] ← save as a txt concatenation of all re-scaled versions of barStruct_k;
    [names2, baridx2] ← save as txt concatenation of all barcodes in barStruct excluding k'th ;
    [mp1, mpI1] ← scamp –window=300 –input_a_file_name=names2 –input_b_file_name=namesBar
     –num_cpu_workers=30 ;
    for j ← 1 to N do
        oS(k, j) ← mp_to_struct(mp1, mpI1, baridx2, stridx, w, sF, barStruct);
    end
end
```

Algorithm 1: Function calc_overlap_mp which calculates pairwise overlaps using the MP algorithm. Our particular implementation is done using $MATLABR2020a$. We set $w = 300$, see Table 3. The function mp_to_struct is shown in Algorithm 2.

```
function : mp_to_struct
Input    : mp1, mpI1, baridx2, stridx, w, sF, barStruct
Output   : oS

for k ← 1 to N do
    for j ← 1 to N do
        if k ≠ j then
            subMP ← mp1(baridx2==j);
            subMPI ← mpI1(baridx2==j);
            oS(k,iy).score ← max(subMP);
            oS(k,iy).pB ← argmax(subMP) ;
            oS(k,iy).pA ← subMPI(oS(k,iy).pB)+2-posstridx ;
            oS(k,iy).or ← sign(stridx(subMPI(oS(k,iy).pB)+1));
            oS(k,iy).bestBarStretch ← sF(abs(stridx(subMPI(oS(k,iy).pB)+1)) ;
        else
            oS(k,iy).score ← nan ;
            oS(k,iy).pB ← nan ;
            oS(k,iy).pA ← nan ;
            oS(k,iy).or ← nan ;
            oS(k,iy).bestBarStretch ← nan ;
        end
    end
end
```

Algorithm 2: Function mp_to_struct, which converts the MP output to a structure.

The local overlap scores use only local information on the overlap between two barcodes. To make use of the all information in the overlap of two barcodes, we define a second score, C_{leftover}, independent of the local score, defined as PCC on the leftover pixels between the two barcodes. This additional score is calculated using Algorithm 3.

```
function : get_leftover_score
Input    : pA,pB,bestBarStretch,orr,barStruct,h
Output   : partialScore,aFul,bFul

lpA ← length(aBar);
lpB ← length(bBar);
st ← min(pA,pB);
stop ← min(lpA-pA+1,lpB-pB+1);
aFul ← bBar(pB-st+1:pB+stop-1);
bFul ← aBar(pA-st+1:pA+stop-1);
aFul(st:st+h-1) ← [];
bFul(st:st+h-1) ← [];
partialScore ← zscore(aFul,1)*zscore(bFul,1)'/length(aFul);
```

Algorithm 3: Function get_leftover_score which calculating the leftover overlap score between two barcodes.

S2.2 Null distribution parameters

Shorter non-matching pairs of barcodes have a higher chance of getting a large PCC score by chance than longer pairs of barcodes. In previous study, we used a p-value based approach to estimate a significance of a PCC score

Table 3: Parameters used in the algorithm

Name	Description	Value
w	minimum overlap	300 (px)
sFstep	length re-scaling step	0.01 (0.025 for validation)
sFmax	max re-scaling	0.1 (0.2 for validation)
numframes	number of frames in a kymograph	up to 20 (timeframes)
scDiffSetting	Allowed scaling factor diff for merging	0.05
pxDifSetting	Allowed pixel difference for merging	50 (px)
$p_{\text{thresh}}^{\text{local}}$	p-value local threshold	0.05
$p_{\text{thresh}}^{\text{leftover}}$	p-value leftover threshold	0.05
α_ν	PCC model constant corresponding to n_{eff}	0.09 (0.085 for real experiment)
α_{N}	PCC model constant corresponding to N_{eff}	0.42 (0.15 for real experiment)
β_ν	local model constant corresponding to n_{eff}	0.085 (0.065 for real experiment)
β_{N}	local model constant corresponding to N_{eff}	0.004 (0.002 for real experiment)

[10], and also of a local overlap score [11]. The p-value approach uses a null model described by the distribution for largest PCC in a set of placement attempts. This distribution has two parameters: ν_{eff}, the effective length of the overlap between the two barcodes, and N_{eff}, which is the effective number of attempts. We here improve on our previous procedure by effectively tabulating the two parameters of the null model distributions for the different scenarios of interest herein. We use the tabulated parameters to estimate constants α_ν, α_N, β_ν, β_N so that they are dependent only on the point spread function of the system, which is fixed by reproducible experimental conditions. We assume that α's relate to the actual length of the overlap, and β's relate to the number of attempts,

$$\alpha_\nu = \frac{\nu_{eff}^{PCC}}{\textit{shortest length}},\ \alpha_N = \frac{N_{\text{eff}}^{PCC}}{\textit{placement attempts}},\ \beta_\nu = \frac{\nu_{\text{eff}}^{local}}{\textit{local length}},\ \beta_N = \frac{N_{\text{eff}}^{local}}{\textit{placement attempts}} \tag{2}$$

where we consider two scenarios for ν_{eff} and N_{eff}: PCC (sliding Pearson Cross Correlation of a short barcode along a long barcode [10]) and local [11].

In Fig S1, we fit the parameters for PCC approach. To this end, we generate sets of 100 synthetic barcodes for a range of lengths L_A and L_B, where $L_A \leq L_B$. We calculate sliding PCC values and get the 100 best PCC scores for each pair of lengths l_A, l_B, $l_A \in L_A$ and $l_B \in L_B$. We then tabulate the parameters ν_{eff} and N_{eff} using maximum likelihood fit to the the functional form [10]. In Fig S1 (A), we show that $\frac{\nu_{\text{eff}}}{L_A}$ can be assumed to be linear, and in Fig S1 (B) we show that $N_{\text{eff}}/(2 \cdot L_B)$ can be assumed to be linear. (C) and (D) shows parameter heatmaps, and (E) shows example EVD histogram fit. Note that we choose $\alpha_\nu = 0.09$ and $\alpha_N = 0.42$ as final values for the selected point spread function conditions. In Fig S2 we similarly fit parameters for local overlap scores. In this case we fix the L_B value and vary L_A and w. Note that we select final values $\beta_\nu = 0.085$, and $\beta_N = 0.004$.

For the real experiments, we use a dataset of experiments using the same experimental procedure, but of another *E.coli* genome. Similarly to the synthetic data, the results of this fitting procedure are presented in Fig S3 and Fig S4, with the selected constant values in Table 3.

S2.3 Positional distance

To validate the alignment quality between assembled barcodes and the reference genome (see Figures 4 and 6 in the main text), we calculate the **positional distance** between each placed barcode and its corresponding location in the ground truth. This distance is computed as follows:

1. Select one barcode as an anchor (e.g. barcode 1) and match it to its known ground-truth position.
2. Compute the relative offset (in pixels) between the anchor and another barcode (e.g. barcode 2) in the assembly.
3. Adjust this offset to account for any length re-scaling.
4. Apply the offset to the anchor's ground truth position to compute the predicted position of the second barcode.
5. The positional distance is the absolute difference between the predicted and actual ground truth start positions of the second barcode.

Supplementary Figures

We here present further figures which serves as support for the figures and conclusions in the main text.

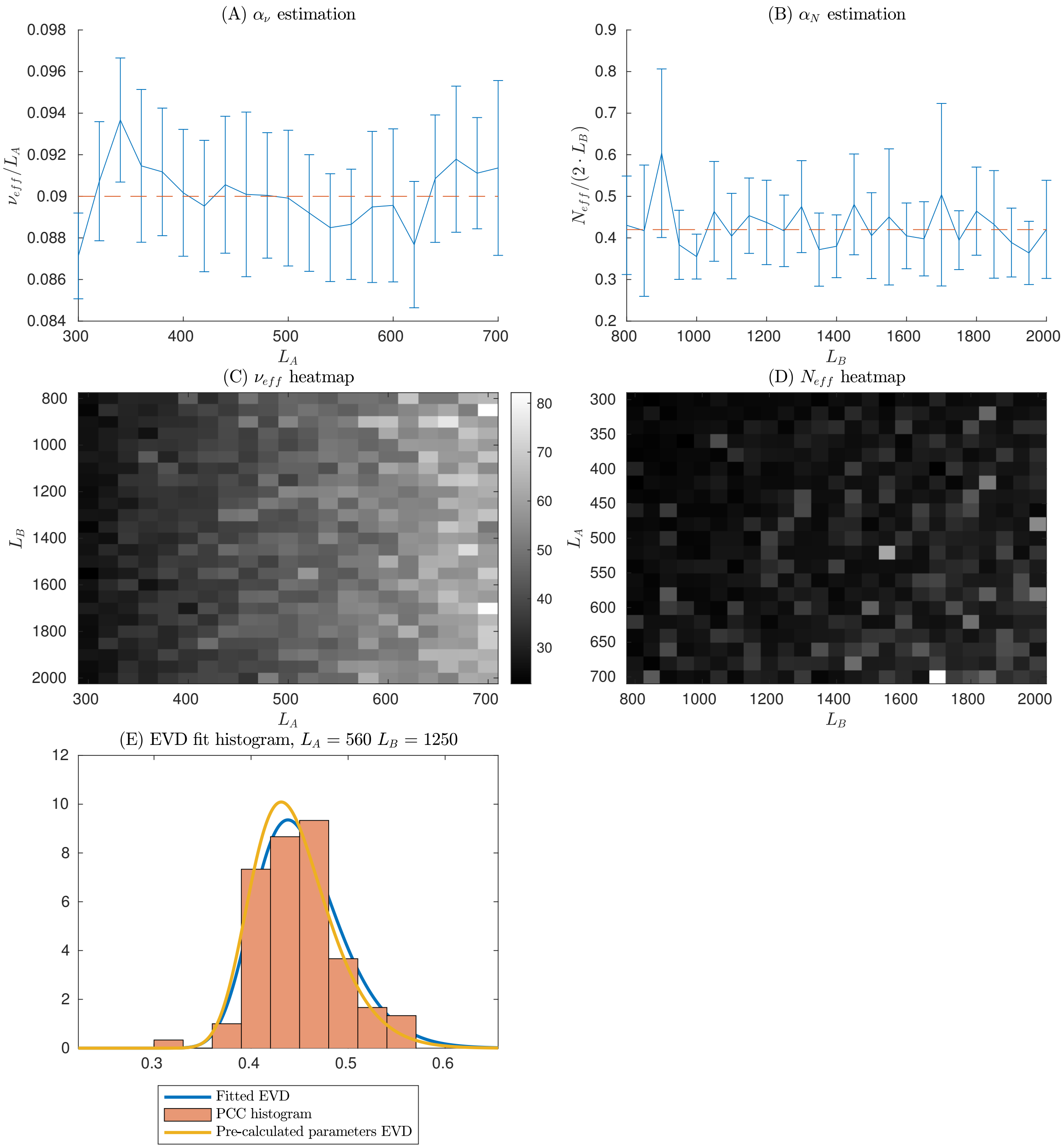

Figure S1: **Null-model parameter estimates for the simple PCC approach.** (A) α_v for a range of L_A values. Each data point is an average over L_B values. (B)) α_N for a range of L_B values. Each data point is an average over L_A values. (C) Heatmap for v_{eff} over the range of L_A, L_B parameter values. (D) Heatmap for N_{eff}. (E) Null-model histogram with associated fit. The pre-calculated model used constants α_ν and α_N, as listed in Table 3.

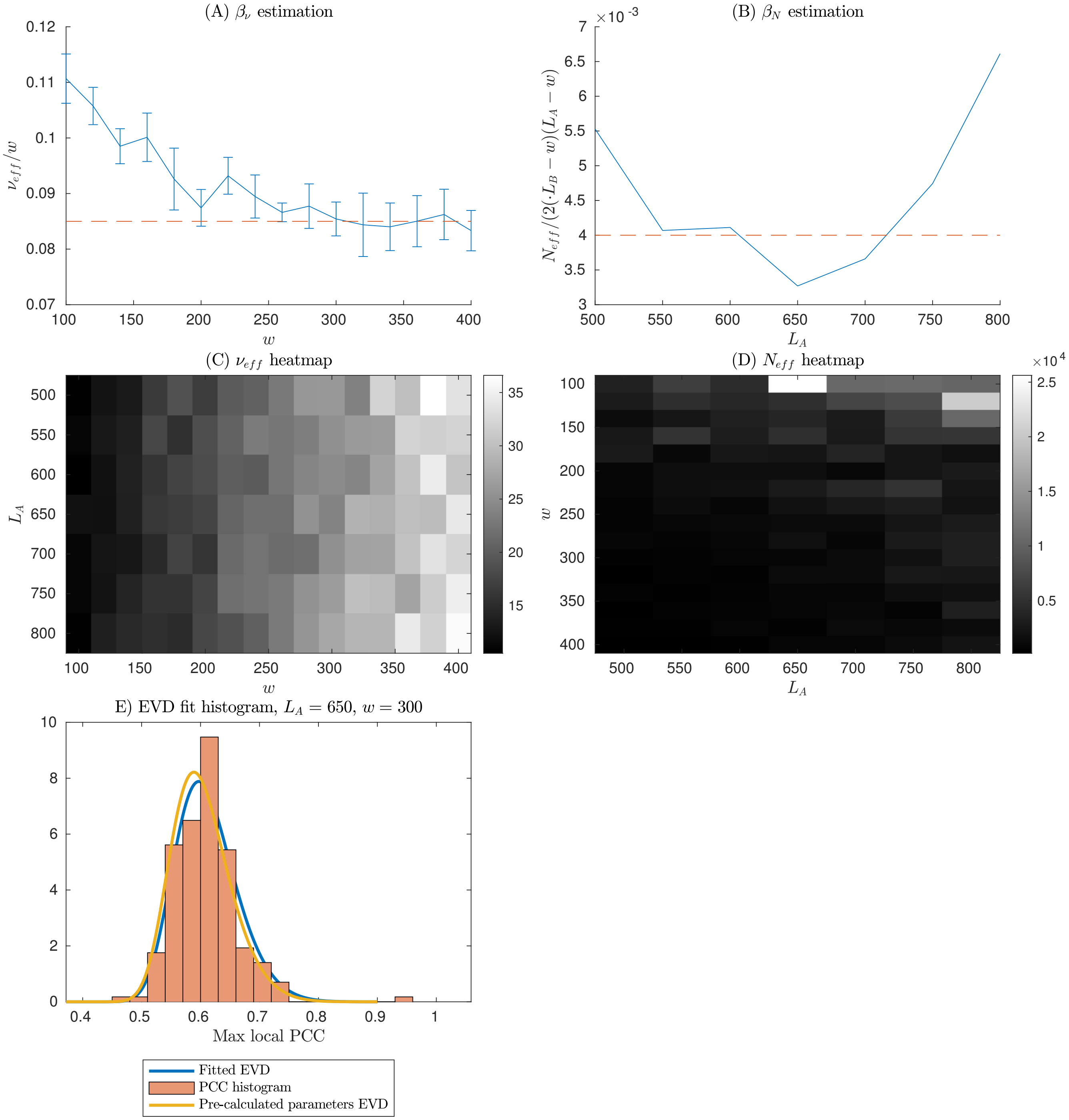

Figure S2: **Null-model parameter estimates for the local alignment score.** (A) β_v estimation. We estimate $\beta_v = v_{eff}/w$. (B) β_N estimation for a fixed $w = 300$. (C-D) Parameter heatmaps. (E) Null-model histogram with associated fits. Pre-calculated model used constants β_ν and β_N, as listed in Table 3.

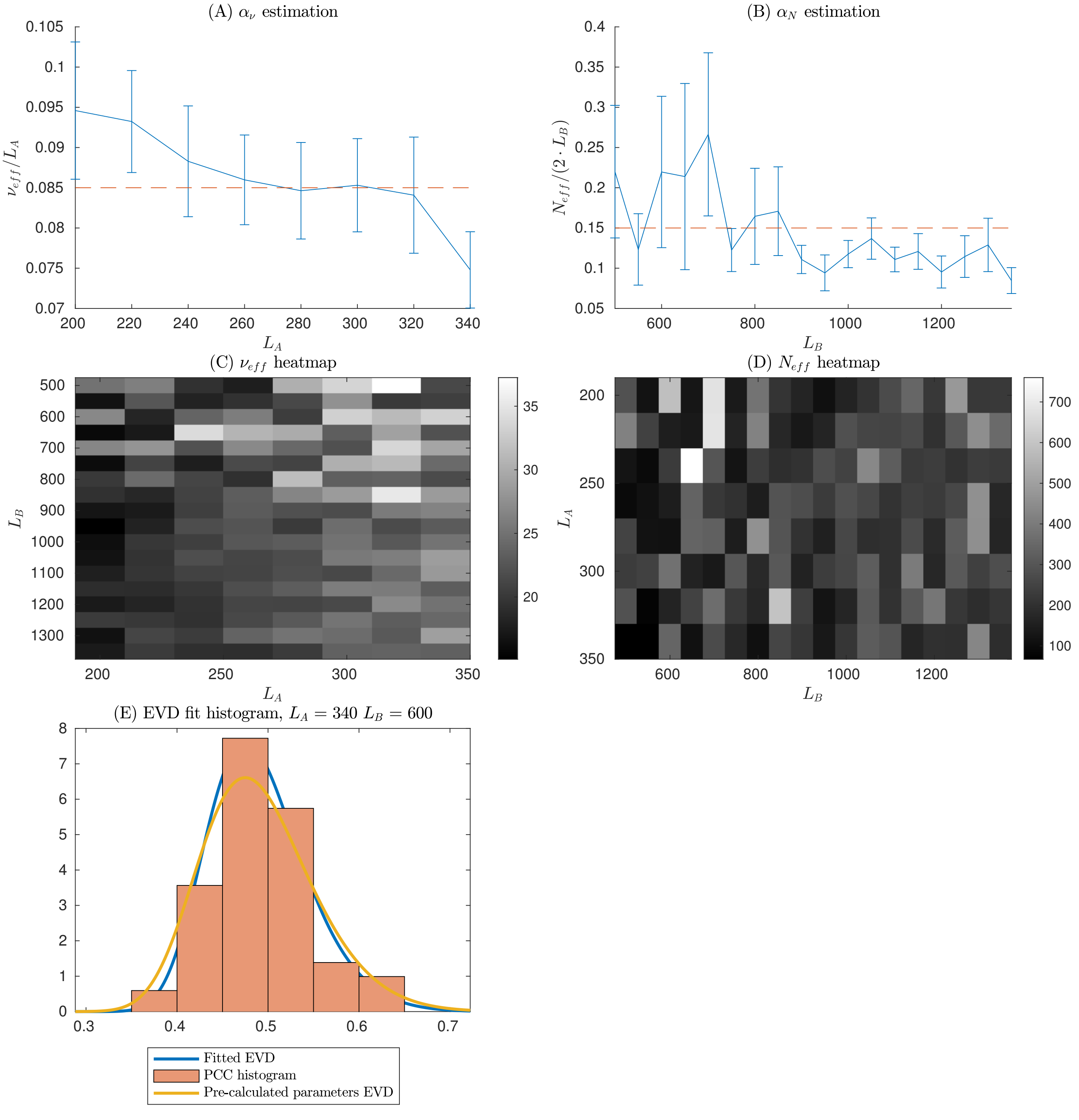

Figure S3: **Parameter fits for simple PCC model of real experiment** (A) α_v for a range of L_A values. Each data point is an average over L_B values. (B)) α_N for a range of L_B values. Each data point is an average over L_A values. (C) Heatmap for v_{eff} over the range of L_A, L_B parameter values. (D) Heatmap for N_{eff}. (E) Null-model histogram with associated fit. Pre-calculated model used constants α_ν and α_N, as listen in Table 3.

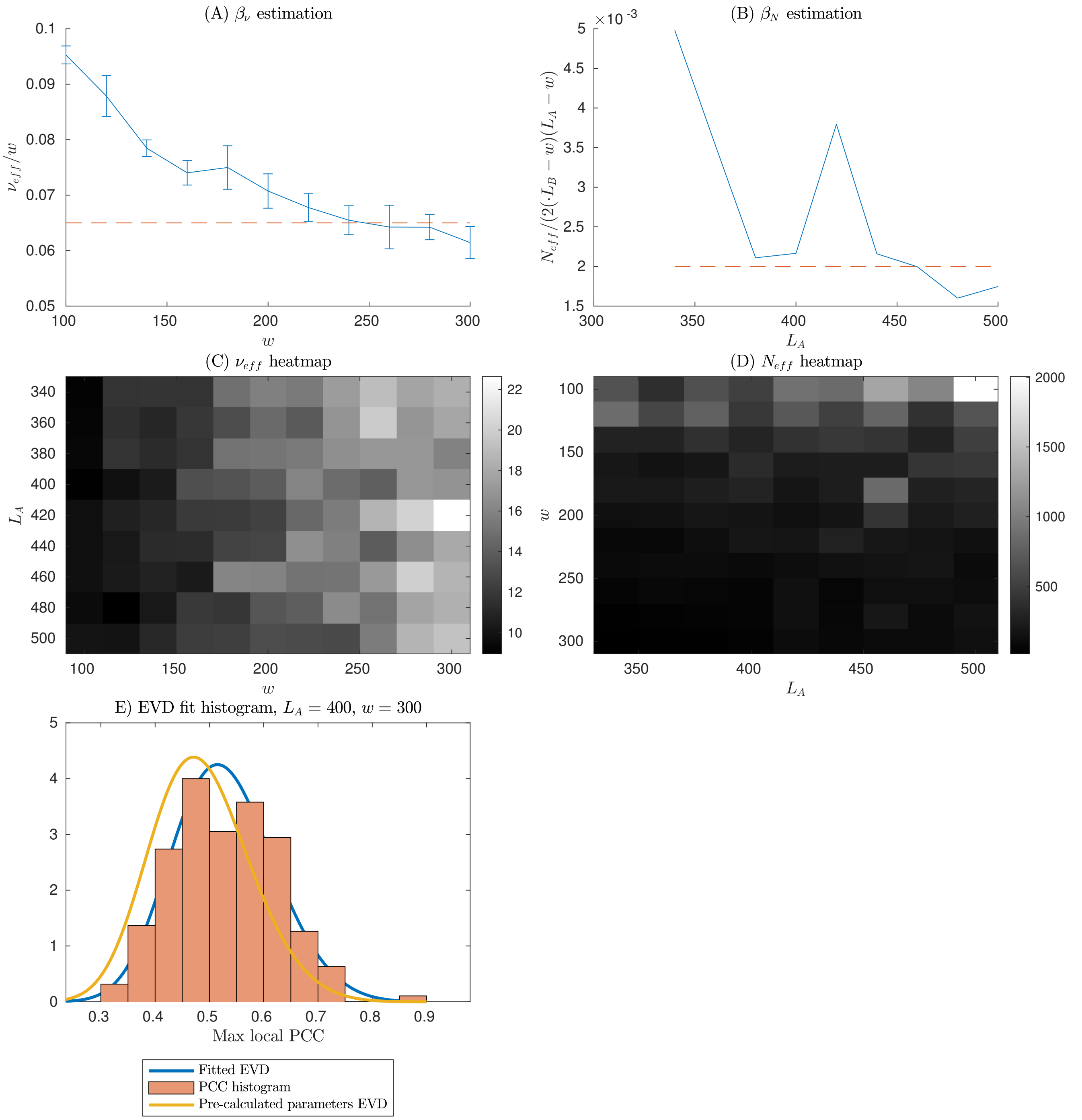

Figure S4: **Parameter fits for null distribution of the local alignment score of real experiment.** (A) β_v estimation. We estimate $\beta_v = v_{eff}/w$. (B) β_N estimation for a fixed $w = 300$. (C-D) Parameter heatmaps. (E)Null-model histogram with associated fit. Pre-calculated model used constants β_ν and β_N, as listed in Table 3.

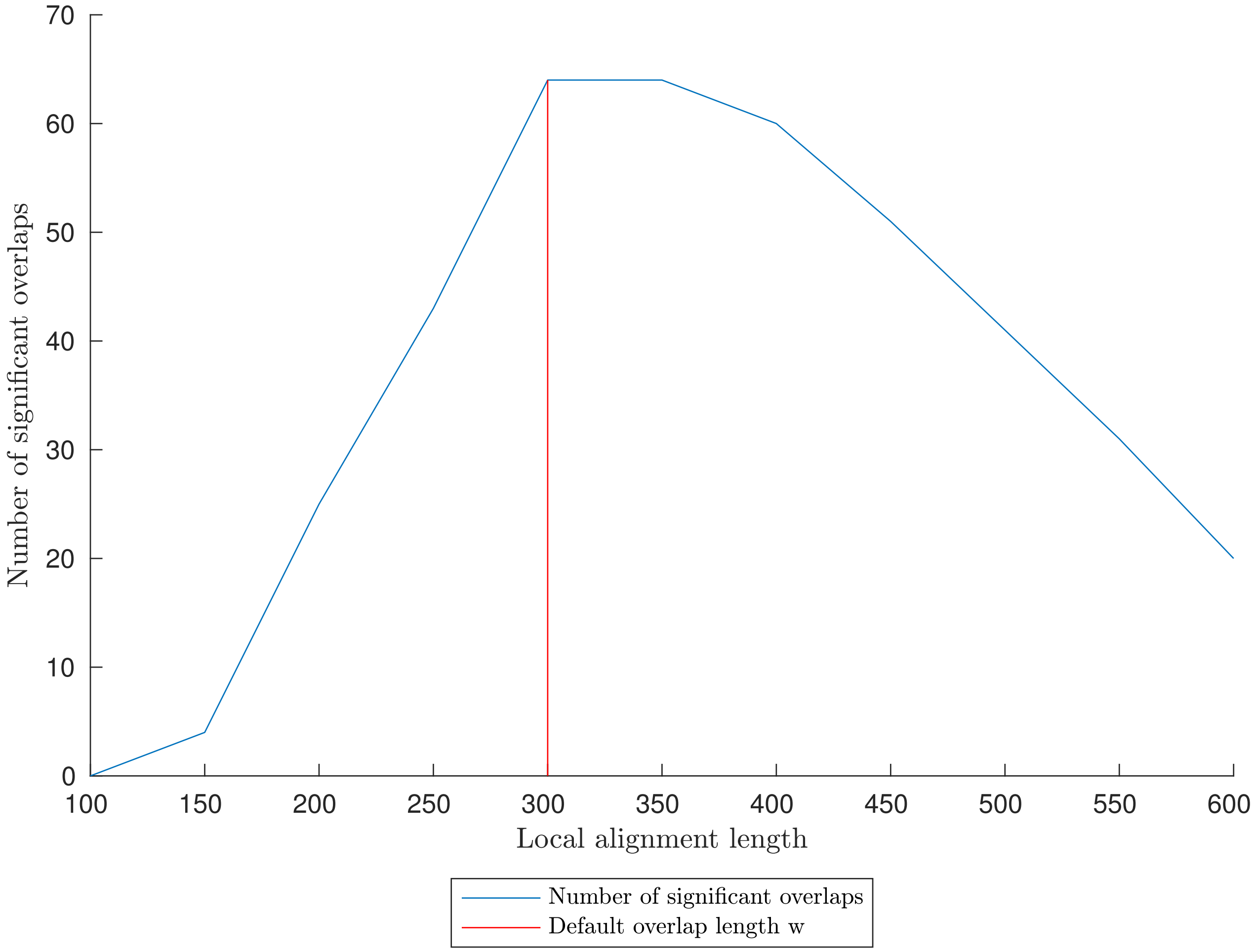

Figure S5: **Parameter choice for minimum overlap length** We calculate a number of significant overlaps for a range of minimum overlap lengths for synthetic barcodes of fragmented synthetically generated genome. Here we considered 50 barcodes and overlap lengths from 100 to 600 px. Note that for our default overlap length w the number of significant overlaps is highest in our analyzed example.

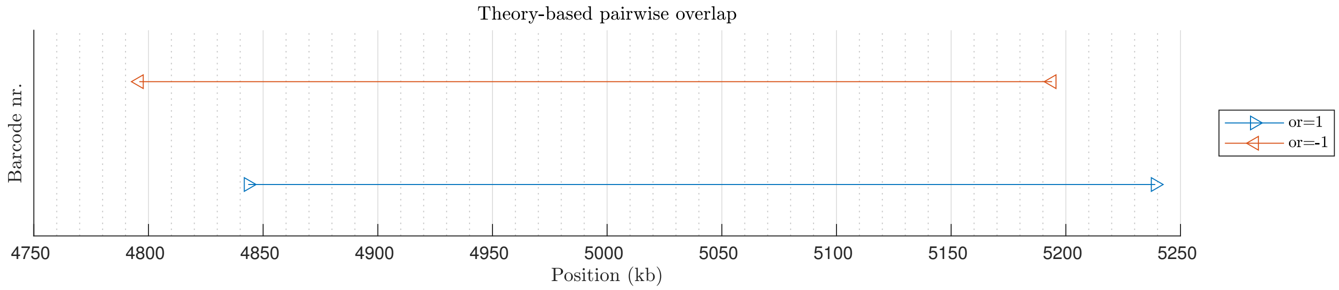

Figure S6: **An example of reference-based pairwise overlap.** Positional placement along the reference for the two barcodes in the main text, Fig. 4 (A).

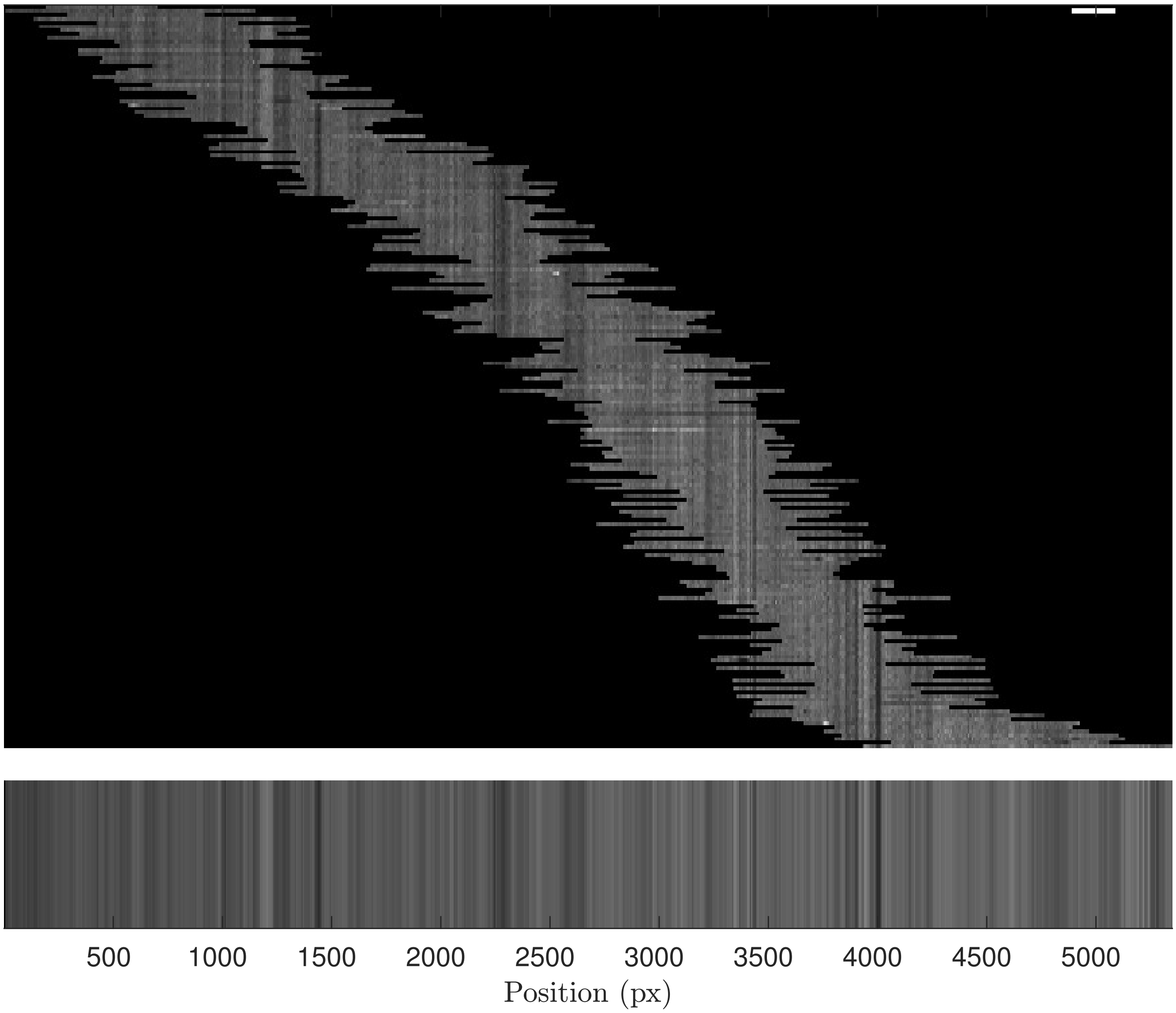

Figure S7: **Block representation (1).** Full block representation for block island (1) from Fig. 6 in the main text with corresponding consensus barcode. The scale bar is 100 kbps.

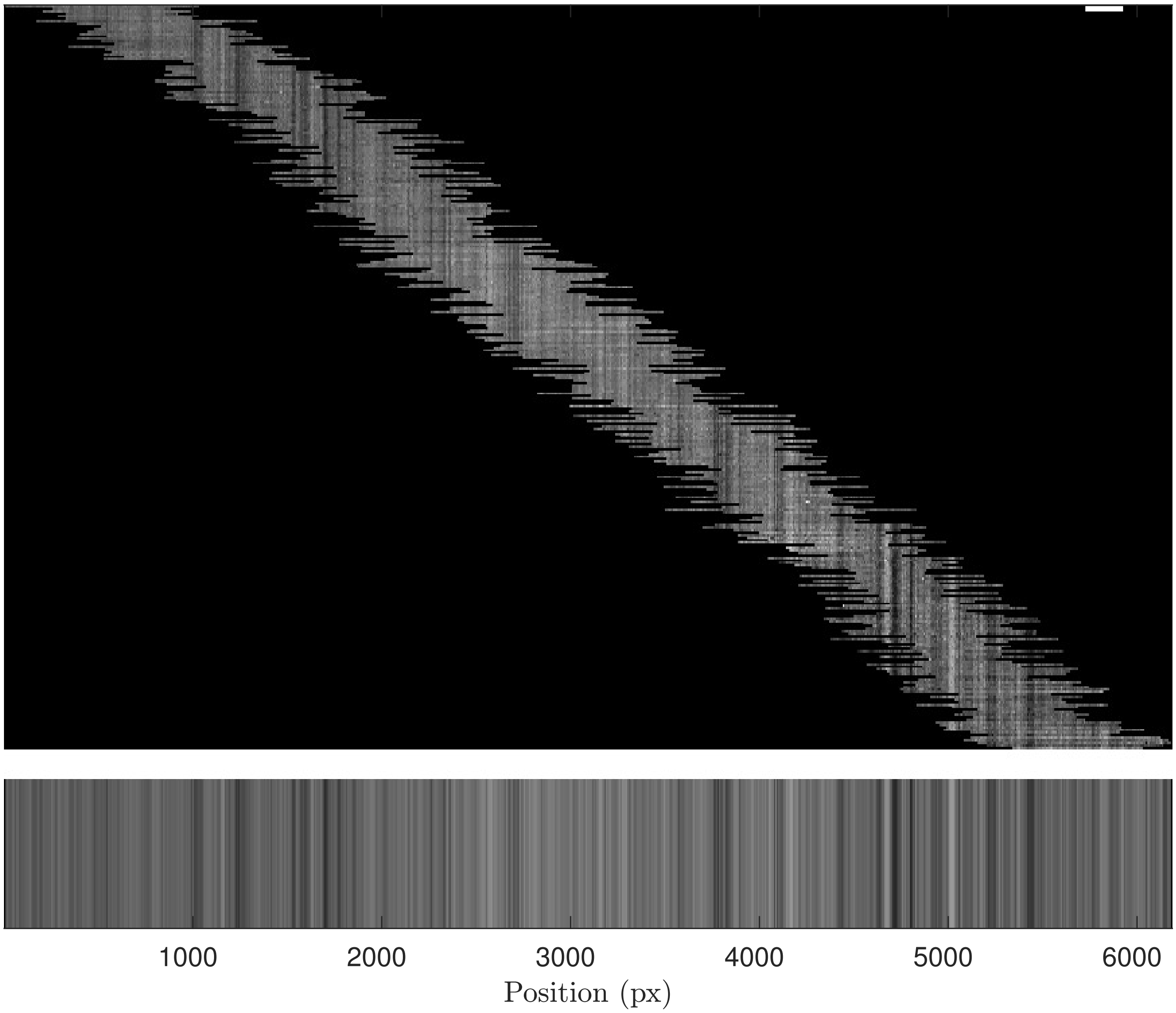

Figure S8: **Block representation (2).** Full block representation for block island (2) from Fig. 6 in the main text with corresponding consensus barcode. The scale bar is 100 kbps.

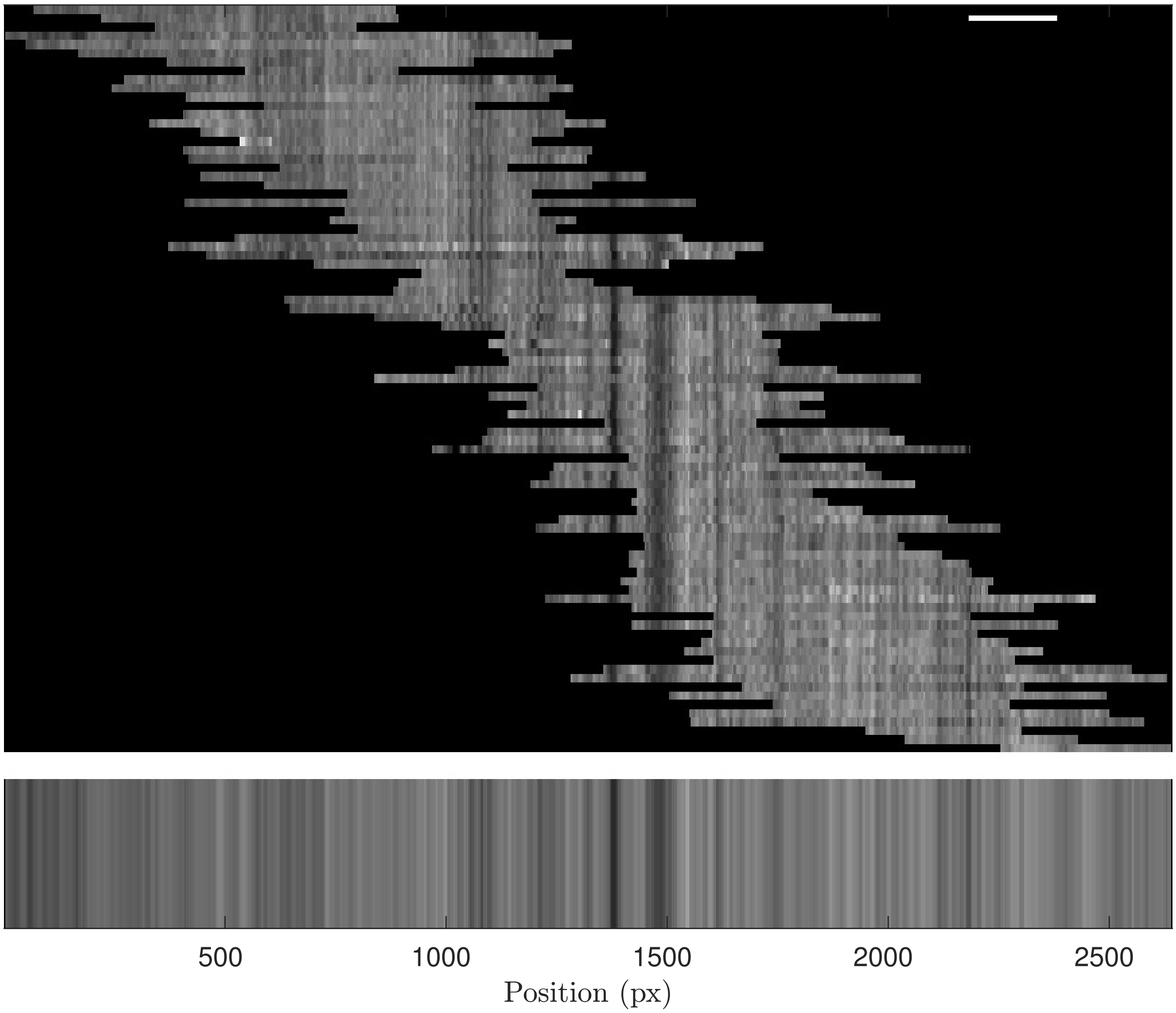

Figure S9: **Block representation (3).** Full block representation and consensus barcode for block island (3) from Fig. 6 in the main text. The scale bar is 100 kbps.

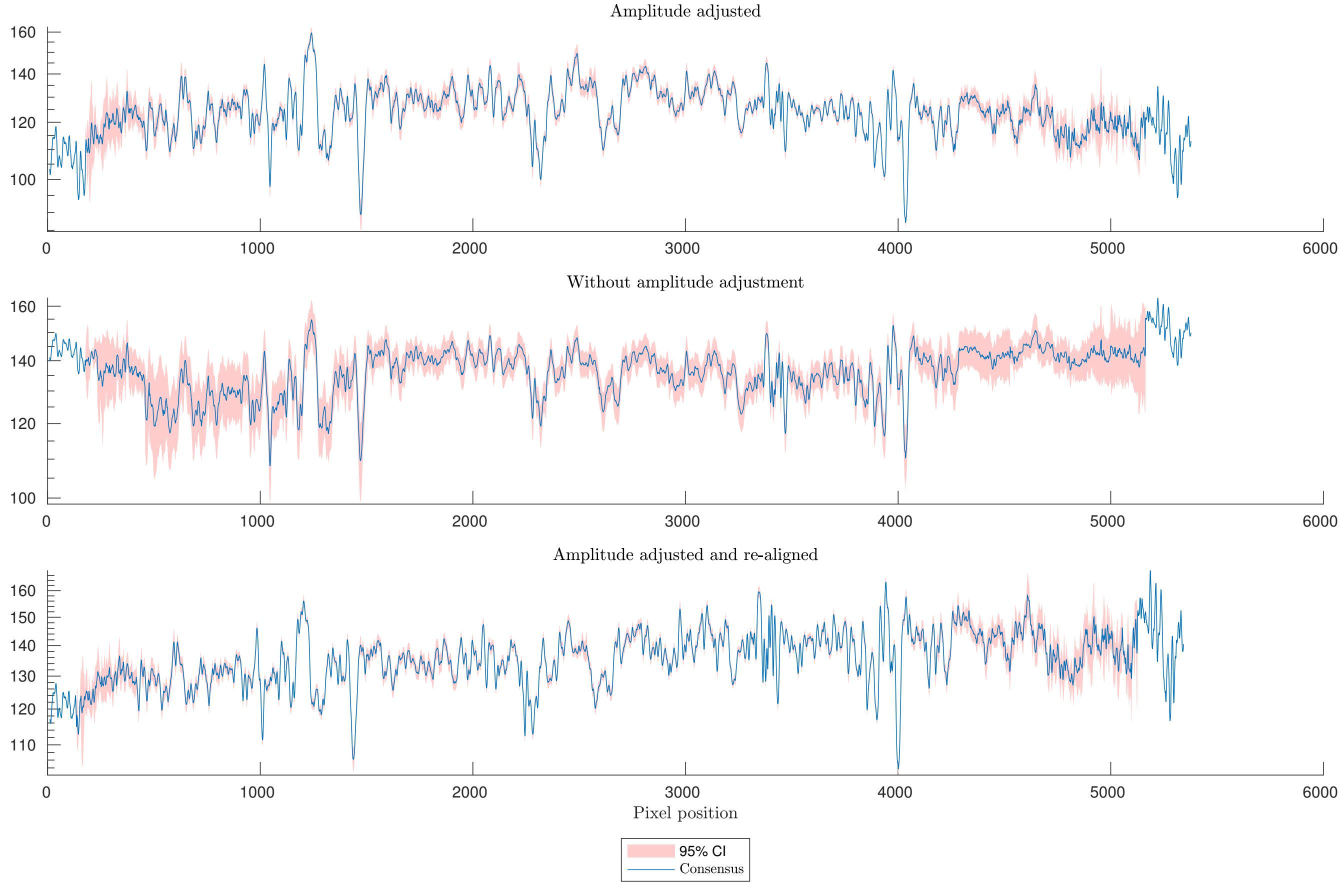

Figure S10: **Comparing three different methods for generating a consensus barcode.** In this plot we consider the consensus for barcode island 1 from Fig. 6 in the main text. (Top) Average of the barcode island with only amplitude adjustment procedure. (Middle) Simple average, no amplitude and no realignment. (Bottom) Both amplitude and realignment. Notice that the variance in amplitude along the consensus barcode is smallest for bottom plot, i.e. for the consensus procedure described in the main text.

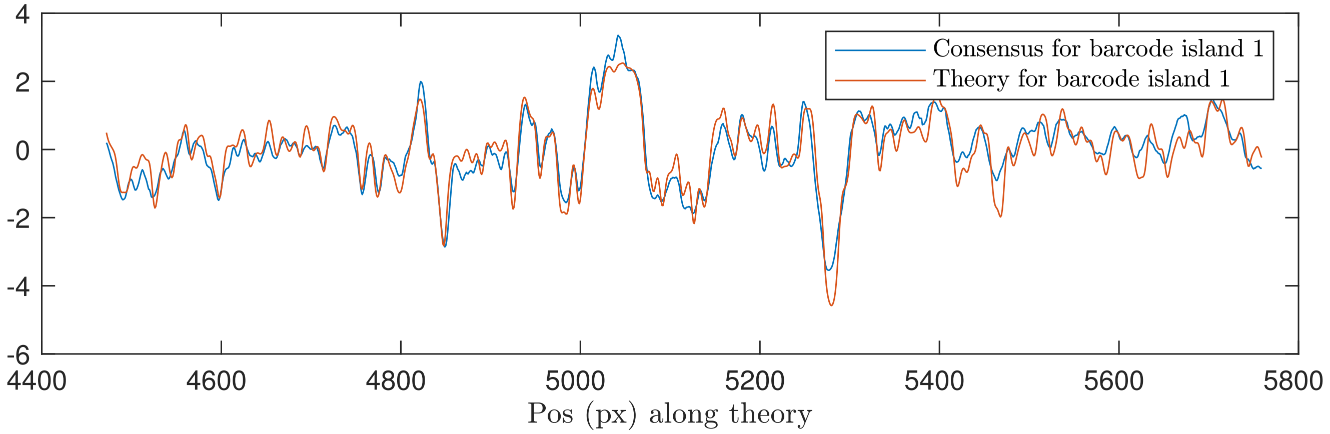

Figure S11: **Comparison of consensus of barcode island vs theoretical barcode.** Zoomed in consensus from Fig. 7 is compared to the associated theoretical barcode.

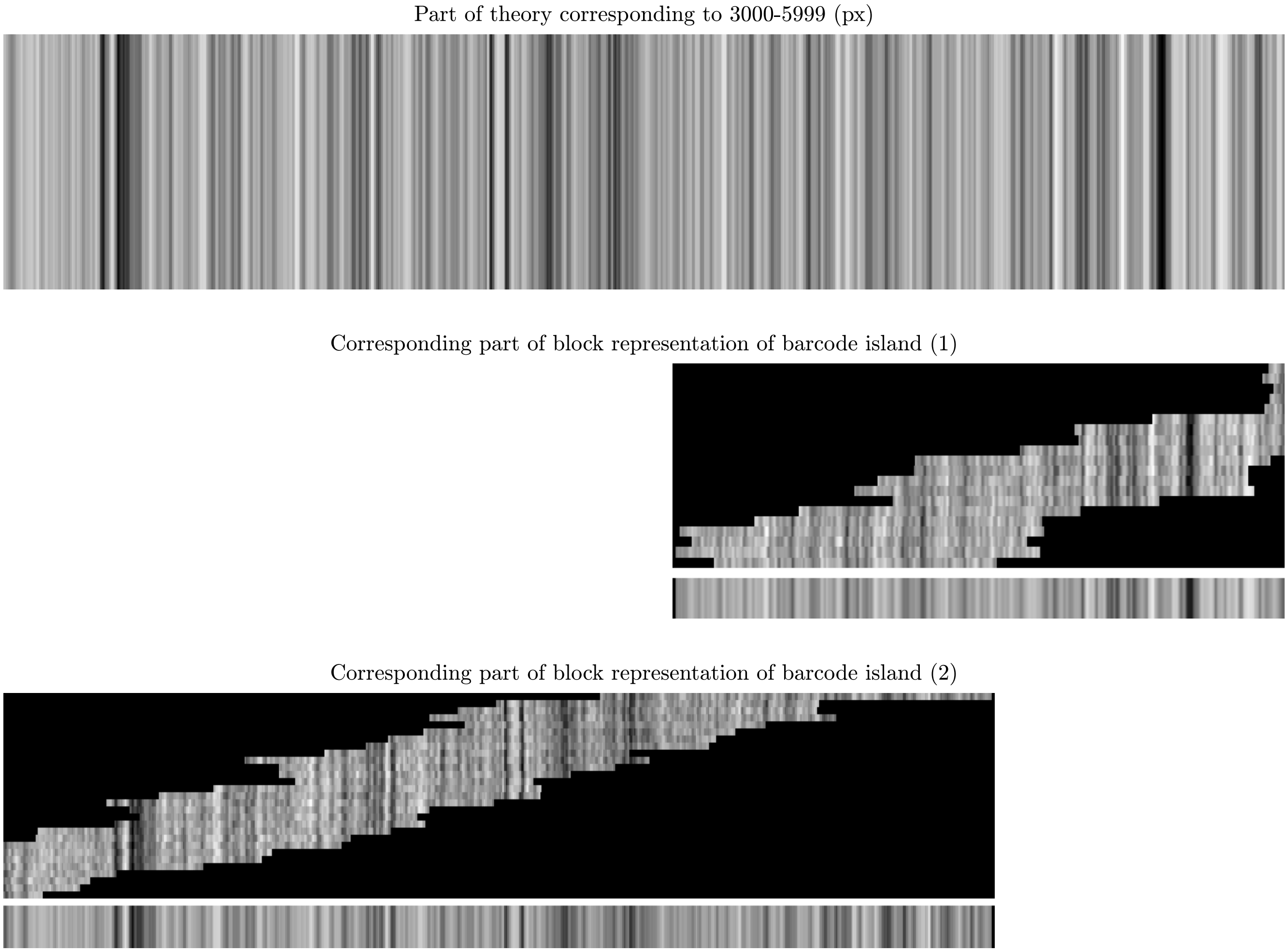

Figure S12: **Zoom-in to the part of the t the two barcode islands overlap.** Comparison of the overlapping region from barcode islands 1 and 2 from Fig. 5 in the main text. (Top) Zoom-in to the theory barcode (Middle) Part of the barcode island 1 overlapping the specific part of the theory barcode. (Bottom) Part of barcode island 2 overlapping the specific part of the theory barcode.

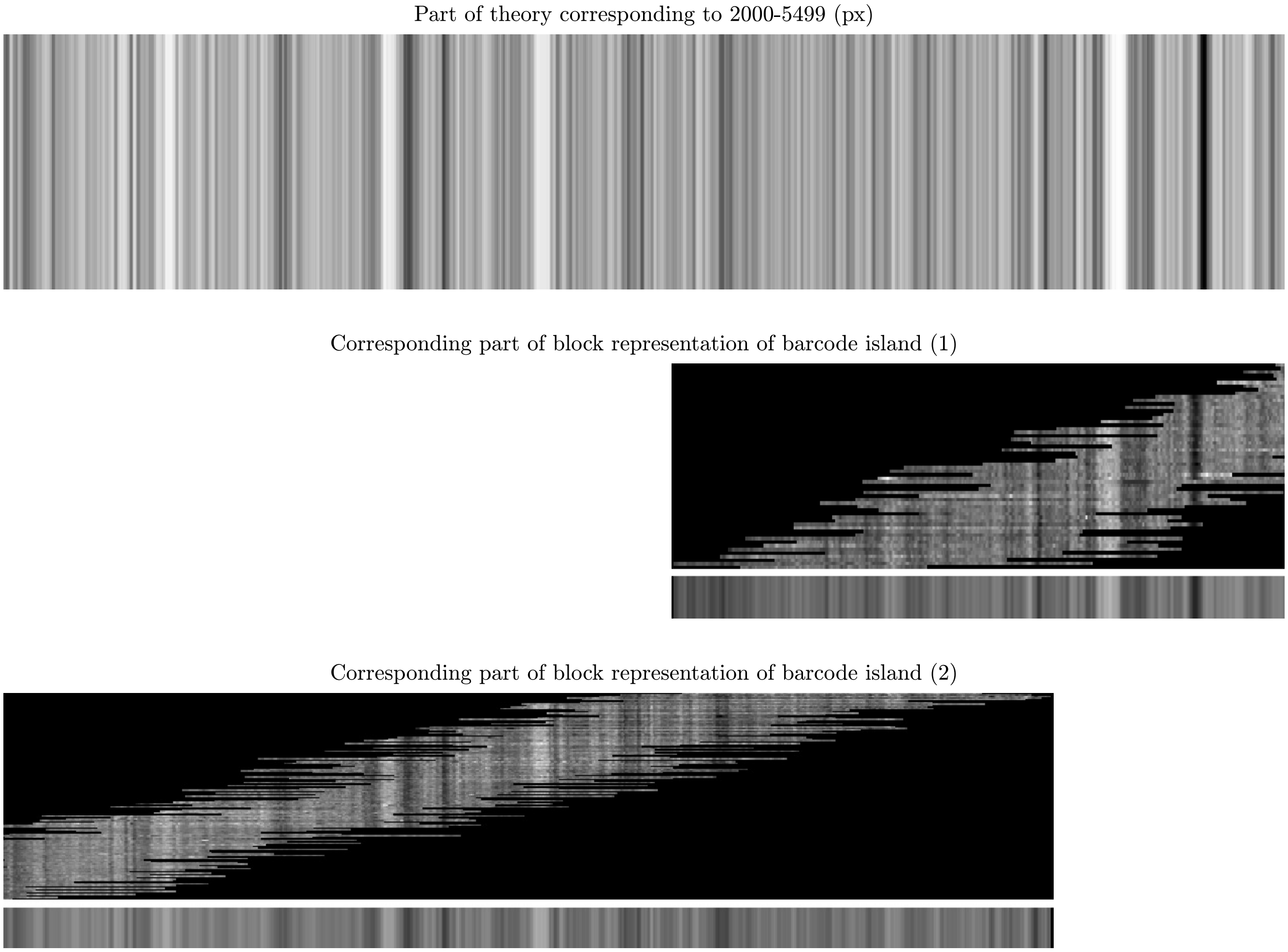

Figure S13: **Zoom-in to the part of the theory that the two barcode islands overlap.** Comparison of the overlapping region from barcode islands 1 and 2 from Fig. 6 in the main text. (Top) Zoom-in to the theory barcode (Middle) Part of the barcode island 1 overlapping the specific part of the theory barcode. (Bottom) Part of barcode island 2 overlapping the specific part of the theory barcode.

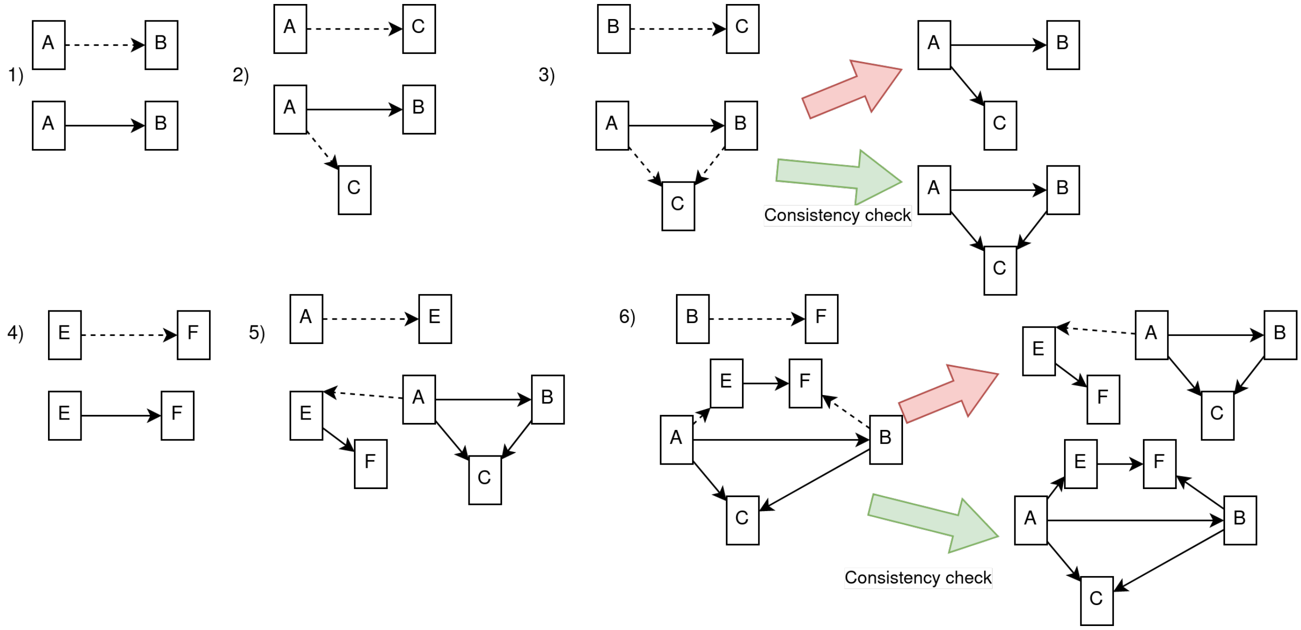

Figure S14: **An example of step 3 in the DOGMA pipeline (see Methods, Analysis pipeline.** 1) Both reference (A) and query (B) are new, so we create a new barcode. 2) A new query (C), but the same reference. We keep this in a merge table (visually, a dotted arrow). 3) The same query (C) appears a second time, and this time we merge it into the barcode (dotted arrows become filled arrows). 4) A new reference and query appears, we create a new barcode. 5) A new query appears (E), this time we create a dashed arrow between the two barcodes. 6) Another new query appears (F), and this time we merge two barcodes into a single barcode island.

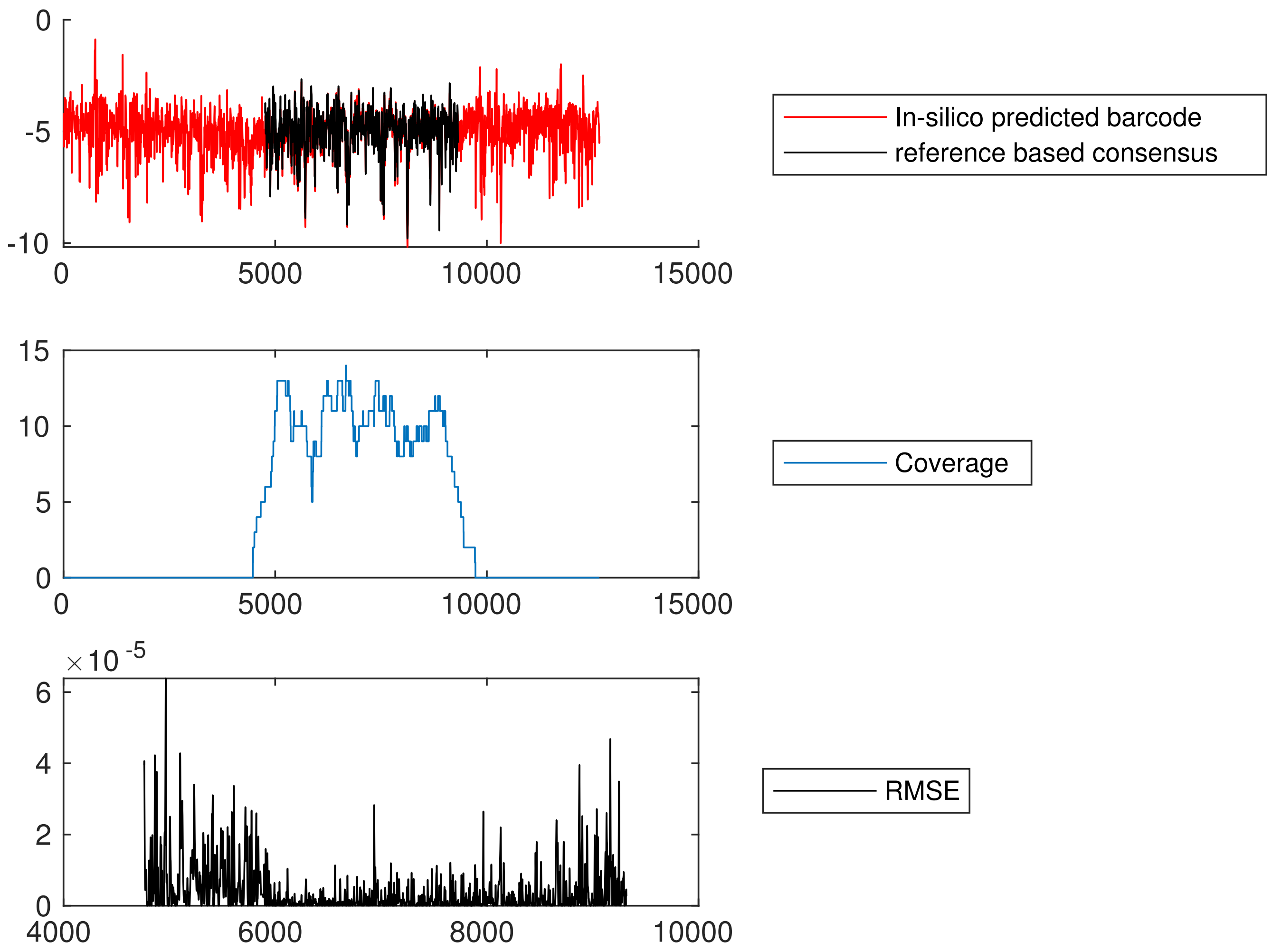

Figure S15: **Mapping individual barcodes of a barcode island graph to the synthetic theory.** Barcodes correspond to barcode island 1 in Figure 5 in the main text. (Top) Reference based consensus is z-normalized and plotted on top of the z-normalized theoretical barcode. (Middle) Coverage along the pixel positions of the theoretical barcode (Bottom) Root mean square error.

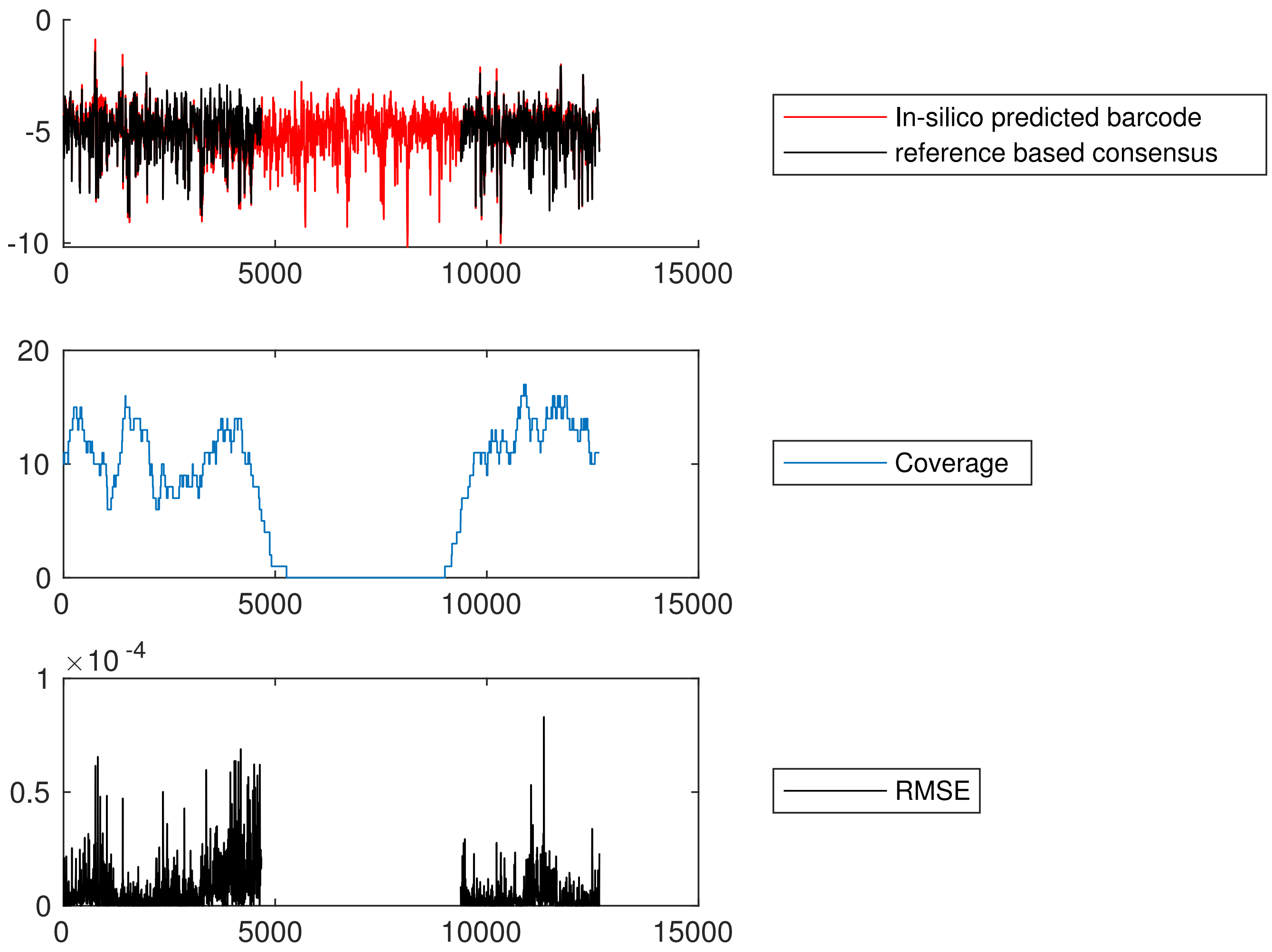

Figure S16: **Mapping individual barcodes of a barcode island graph to the synthetic theory.** Barcodes correspond to barcode island 2 in Figure 5 in the main text. (Top) Reference based consensus is z-normalized and plotted on top of the z-normalized theoretical barcode. (Middle) Coverage along the pixel positions of the theoretical barcode (Bottom) Root mean square error.

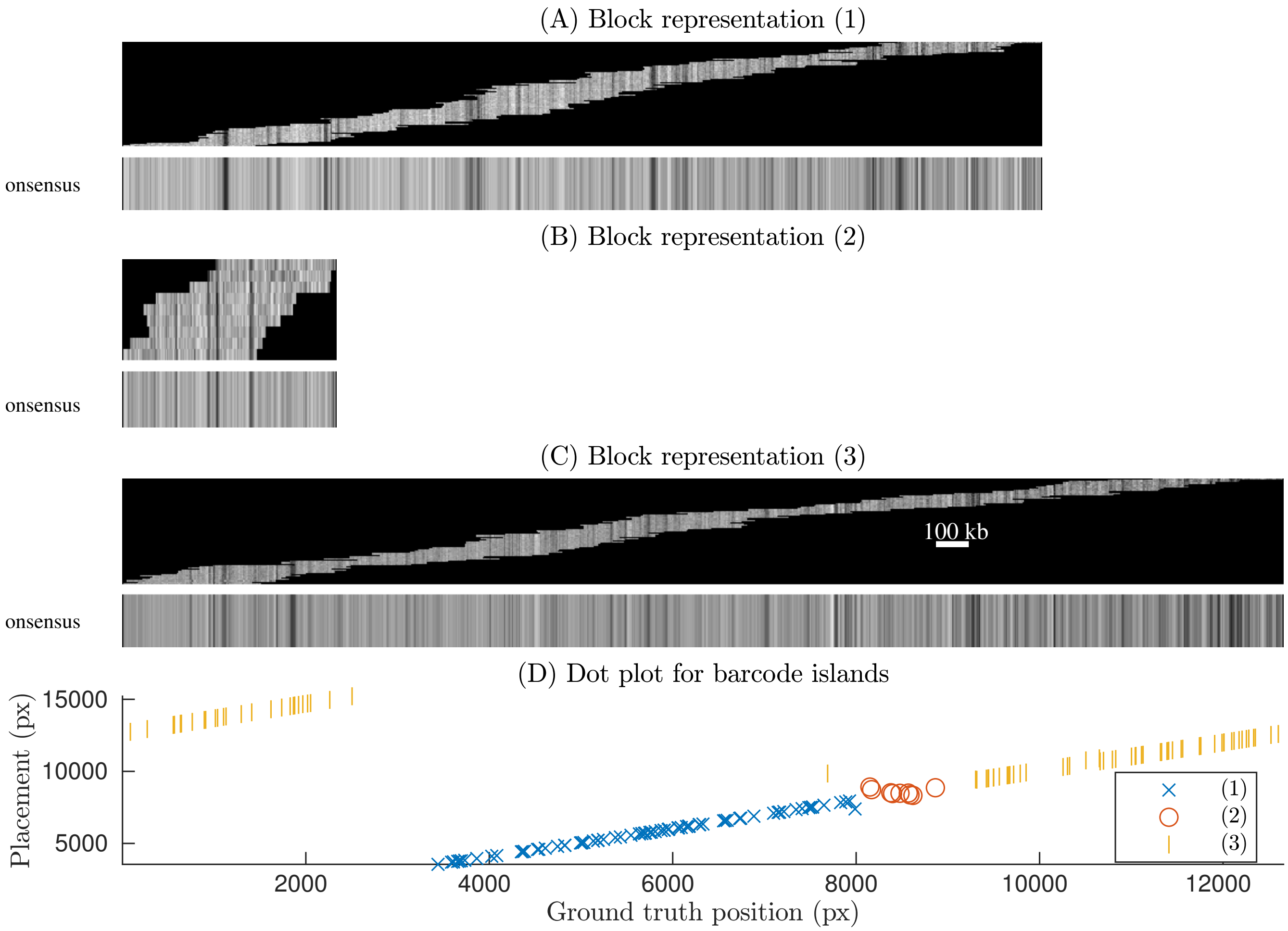

Figure S17: **Barcode islands and corresponding dot plot validation for synthetic barcodes.** Corresponds to data in the blue square in Fig. 3 in the main text. (A-B) Block representation for barcode islands (1-2) along with the associated consensus barcodes. (C) Corresponding dot plot. The x-axis is the starting position along the theoretical barcode, and the y-axis is the starting position along the barcode island after alignment to the theoretical barcode. Note that the two islands cover two distinct (but possibly overlaping) regions along the chromosome. In this example the dataset with synthetic-noise variance ratio = 0.75 and # barcode = 175 is considered, see Fig. 3 in the main text.

References

[1] Müller V, Nyblom M, Johnning A, Wrande M, Dvirnas A, Kk S, Giske CG, Ambjornsson T, Sandegren L, Kristiansson E, Westerlund F. Cultivation-free typing of bacteria using optical DNA mapping. ACS infectious diseases. 2020 Apr 15;6(5):1076-84.

[2] Müller V, Dvirnas A, Andersson J, Singh V, Kk S, Johansson P, Ebenstein Y, Ambjörnsson T, Westerlund F. Enzyme-free optical DNA mapping of the human genome using competitive binding. Nucleic acids research. 2019 Sep 5;47(15):e89-.

[3] Persson F, Tegenfeldt JO. DNA in nanochannels—directly visualizing genomic information. Chemical Society Reviews. 2010;39(3):985-99.

[4] Dvirnas, A. & Lin, Y.-L. albertasdvirnas/lldev: lldev.v.0.5.3 (0.5.3). Zenodo. 2021. https://doi.org/10.5281/zenodo.5718208.

[5] Nyblom, M., Johnning, A., Frykholm, K. et al (2023). Strain-level bacterial typing directly from patient samples using optical DNA mapping. COMMUNICATIONS MEDICINE, 3(31). http://dx.doi.org/10.1038/s43856-023-00259-z

[6] Nordanger H. Gene-ID Using Simultaneous DNA Barcoding and Enzymatic Labeling.

[7] Reisner W, Pedersen JN, Austin RH. DNA confinement in nanochannels: physics and biological applications. Reports on progress in physics. 2012 Sep 13;75(10):106601.

[8] Müller V, Dvirnas A, Andersson J, Singh V, Kk S, Johansson P, Ebenstein Y, Ambjörnsson T, Westerlund F. Enzyme-free optical DNA mapping of the human genome using competitive binding. Nucleic acids research. 2019 Sep 5;47(15):e89-.

[9] Zimmerman, Zachary, et al. "Matrix Profile XIV: Scaling Time Series Motif Discovery with GPUs to Break a Quintillion Pairwise Comparisons a Day and Beyond." Proceedings of the ACM Symposium on Cloud Computing. 2019.

[10] Dvirnas A, Pichler C, Stewart CL, Quaderi S, Nyberg LK, Müller V, Kumar Bikkarolla S, Kristiansson E, Sandegren L, Westerlund F, Ambjörnsson T. Facilitated sequence assembly using densely labeled optical DNA barcodes: A combinatorial auction approach. PloS one. 2018 Mar 9;13(3):e0193900.

[11] Dvirnas A, Stewart C, Müller V, Bikkarolla SK, Frykholm K, Sandegren L, Kristiansson E, Westerlund F, Ambjörnsson T. Detection of structural variations in densely-labelled optical DNA barcodes: A hidden Markov model approach. Plos one. 2021 Nov 5;16(11):e0259670.
